# Using a cellulose-complementary oligosaccharide as a tool to probe exposed cellulosic surfaces in cotton fibres and growing plant cell walls

**DOI:** 10.1042/BCJ20240296

**Published:** 2024-09-13

**Authors:** Mahnoor Imran, Lenka Franková, Uzma Qaisar, Stephen C. Fry

**Affiliations:** 1 The Edinburgh Cell Wall Group, Institute of Molecular Plant Sciences, The University of Edinburgh, Edinburgh EH9 3BF, U.K.; 2 School of Biological Sciences, University of the Punjab, Lahore, Pakistan

**Keywords:** cellulose surface accessibility, cotton fibres, hydrogen bonding, microfibrils, oligosaccharides, paper

## Abstract

Cellulosic microfibrils in plant cell walls are largely ensheathed and probably tethered by hydrogen-bonded hemicelluloses. Ensheathing may vary developmentally as hemicelluloses are peeled to enable cell expansion. We characterised a simple method to quantify ensheathed *versus* naked cellulosic surfaces based on the ability to adsorb a radiolabelled ‘cellulose-complementary oligosaccharide’, [^3^H]cellopentaitol. Filter-paper (cellulose) adsorbed 40% and >80% of aqueous 5 nM [^3^H]cellopentaitol within ∼1 and ∼20 h respectively. When [^3^H]cellopentaitol was rapidly dried onto filter-paper, ∼50% of it was desorbable by water, whereas after ∼1 day annealing in aqueous medium the adsorption became too strong to be reversible in water. ‘Strongly’ adsorbed [^3^H]cellopentaitol was, however, ∼98% desorbed by 6 M NaOH, ∼50% by 0.2 M cellobiose, and ∼30% by 8 M urea, indicating a role for hydrogen-bonding reinforced by complementarity of shape. Gradual adsorption was promoted by kosmotropes (1.4 M Na_2_SO_4_ or 30% methanol), and inhibited by chaotropes (8 M urea), supporting a role for hydrogen-bonding. [^3^H]Cellopentaitol adsorption was strongly competed by non-radioactive cello-oligosaccharides (Cell_2–6_), the IC_50_ (half-inhibitory concentration) being highly size-dependent: Cell_2_, ∼70 mM; Cell_3_, ∼7 mM; and Cell_4–6_, ∼0.05 mM. Malto-oligosaccharides (400 mM) had no effect, confirming the role of complementarity. The quantity of adsorbed [^3^H]cellopentaitol was proportional to mass of cellulose. Of seven cottons tested, wild-type *Gossypium arboreum* fibres were least capable of adsorbing [^3^H]cellopentaitol, indicating ensheathment of their microfibrillar surfaces, confirmed by their resistance to cellulase digestion, and potentially attributable to a high glucuronoarabinoxylan content. In conclusion, [^3^H]cellopentaitol adsorption is a simple, sensitive and quantitative way of titrating ‘naked’ cellulose surfaces.

## Introduction

Cellulose [defined chemically as β-(1→4)-d-glucan] is the world's most abundant organic substance [1]. Different preparations of cellulose differ in physical conformation, especially crystallinity, as well as molecular weight and crystallite size [2]. Most cellulose in nature occurs within plant cell walls, where the individual cellulose molecules are tightly bundled in parallel (as polymorph Iβ) to form microfibrils. Microfibrils are the skeleton of the plant cell wall, responsible for its strength and shape, and they critically contribute to the architecture and growth rate of plants [3,4]. Neighbouring cellulose molecules within a microfibril spontaneously self-aggregate owing to their complementarity of shape [5]. Cellulose is therefore insoluble in water.

In the plant cell wall, microfibrils are often largely covered by a sheath of complementary non-cellulosic polysaccharides, especially xyloglucans and heteroxylans [4,6–8]. In the intact cell wall, these hemicelluloses are firmly hydrogen-bonded to cellulosic surfaces and are proposed to serve as tethers, inter-linking adjacent microfibrils [6,9]. This contributes to cell-wall architecture and controls cell expansion (thus plant growth). In addition, it has been suggested that some xyloglucan molecules penetrate the microfibrils [7,10].

It is unclear exactly what proportion of the cellulosic surface of a microfibril is exposed to the surrounding aqueous environment rather than ensheathed in hemicelluloses. This is of interest for several reasons, both plant-biological and biotechnological. One exciting biological situation where cellulose surface exposure may well change is during plant cell expansion (plant growth), when sheathing hemicelluloses are believed to be partially stripped off cellulose surfaces [11] by mechanical stretching, potentially augmented by chaotropic ‘expansin’ proteins [12]. We predict that new naked surfaces of cellulose are thereby created. Cellulose is also of huge biotechnological importance, e.g. in paper, textiles, medical dressings, viscosity enhancers etc. [13–15]. The surface accessibility of commercial cellulose fibres, crucial in determining their desirable properties, is therefore also of interest.

**Figure 1.**
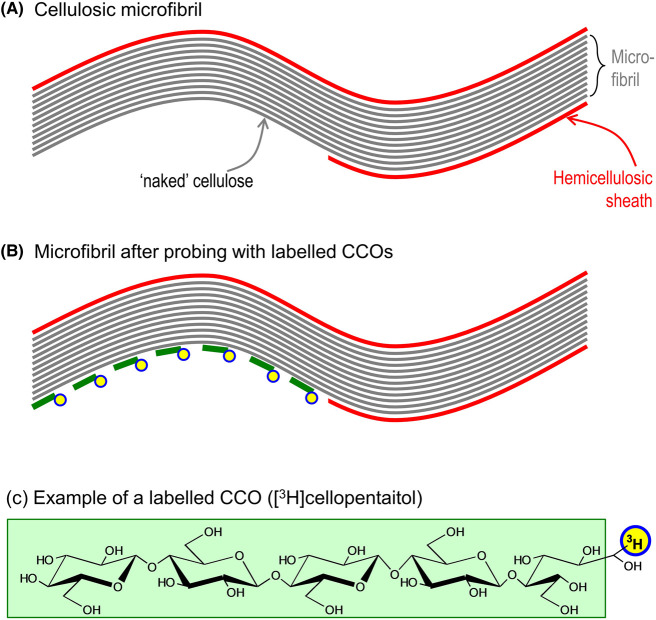
Strategy for detecting exposed surfaces of microfibrils. In this simplified depiction, regions of ‘naked’ cellulose, lacking a hemicellulosic sheath (**A**), are expected to permit hydrogen-bonding of cellulose-complementary oligosaccharides (CCOs) such as cellopentaitol (symbolised in (**B**) as short green lines; full structure shown in (**C**)). To facilitate quantitative detection of bound CCOs, we labelled them with tritium (yellow/blue circles).

One approach to exploring the exposure of surface cellulose is to assay its ability to associate with tritium from [^3^H]water applied as a probe [13]. In the present work, we have tested larger probe molecules (than [^3^H]water) which might provide valuable data on exposed cellulosic areas that are large enough to accommodate hemicelluloses and thus potentially to be tethered. Such probes are termed here ‘cellulose-complementary oligosaccharides’ (CCOs), which may have great potential for assaying the exposed surfaces of cellulose microfibrils in biologically and biotechnologically important specimens. Water-soluble CCOs are expected to hydrogen-bond to ‘naked’ cellulosic surfaces, and they can be detected simply, sensitively and quantitatively if they have been tagged with a suitable ‘label’, and data expressed per gram of total cellulose. The CCO explored here was tritium-labelled cellopentaitol ([^3^H]Cell_5_-ol; Figure 1), which has a high affinity for cellulose, as demonstrated by its immobility on paper chromatography [16]. One exciting future goal is to use this strategy to provide a molecular description of certain major unknowns of plant cell growth.

We characterised the binding of [^3^H]Cell_5_-ol to various sources of cellulose, including filter-paper and cotton fibres from three species of *Gossypium* differing in fibre length. Where available, crystallinity index (CI) values of the types of cellulose used are listed in the Materials and Methods. Cotton ‘fibres’ [botanically, ovular epidermal hair cells] typically contain ∼90% cellulose by dry weight plus much smaller amounts of non-cellulosic cell-wall-matrix polysaccharides (especially pectins) [17,18]. Cotton is the starting raw material for a wide range of products, including textiles, edible oil, paper, livestock feed, medicinal products, and many more [19,20]. We compared the cellulose from three species of *Gossypium* differing in the ‘staple’ rating of their fibres (long-staple cotton having longer, stronger and more uniform fibres): *Gossypium arboreum*, *Gossypium hirsutum* and *Gossypium barbadense* (short, long and extra-long staple respectively). In addition, we tested four transformants of *G. hirsutum* expressing the *WRI1* gene of *G. barbadense* under a fibre-specific promoter, which showed more twisted fibres as compared with the wild-type. WRINKLED1 (WRI1) is a transcription factor of the AP2/EREBP class, which share an AP2 DNA-binding domain [21]. WRI1 regulates the expression of genes involved in fatty acid metabolism and glycolysis [22,23]. Directing the expression of *WRI1* in the fibres through a fibre-specific promoter can enhance the carbon flow towards sugar biosynthesis pathways and play role in fibre development [24,25]. Therefore we were interested to explore the fibre architecture in *WRI1* transformant lines as well as in three species of cotton possessing noticeably different fibre surface characteristics and morphology.

## Results

### Affinity of cellopentaose and cellopentaitol for cellulose, as judged by paper chromatography

To investigate whether [^3^H]Cell_5_-ol [i.e. borotritiide-reduced cellopentaose (Cell_5_)] could be used as a CCO, we tested its behaviour on paper chromatography, Whatman No. 1 paper being almost pure cellulose; for comparison, non-reduced Cell_5_ and several related sugars were also tested (Supplementary Figure S1). It has been shown previously that cellohexaose, xylohexaose and mannohexaose migrate considerably faster than the corresponding reduced oligosaccharides in conventional paper chromatography solvents (solvents c and d in Supplementary Figure S1; cf. Figure 4 of [16]). We confirmed this trend for [^3^H]Cell_5_-ol versus non-radioactive Cell_5_ in solvent (c). The trend was extreme when water was used as the main chromatography solvent, whether at low or high pH (10% acetic acid or 10% pyridine respectively). In conclusion, [^3^H]Cell_5_-ol binds avidly to cellulose and is a promising candidate to serve as a CCO.

### Kinetics of adsorption/desorption of a CCO to/from four commercial celluloses

**Figure 2.**
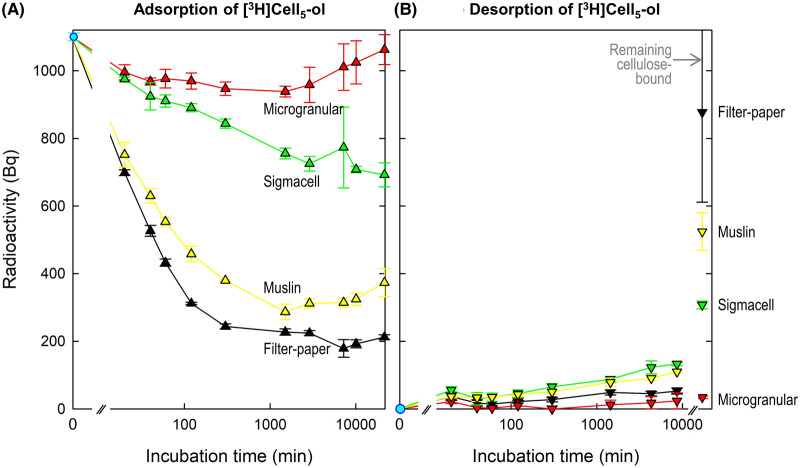
[^3^H]Cell_5_-ol rapidly adsorbs to certain celluloses and slowly desorbs on washing. (**A**) Duplicate portions (90 mg) of each type of cellulose were suspended in 5 ml of 100 mM acetate buffer (Na^+^, pH 4.75, containing 0.5% chlorobutanol) with [^3^H]Cell_5_-ol (1100 Bq; final concentration 5 nM) and incubated with shaking at 20°C. At intervals, duplicate 100-µl aliquots of the solution were assayed for remaining soluble [^3^H]Cell_5_-ol (counting efficiency 33%). (**B**) After 15 days, the cellulose was recovered from the suspension, rinsed briefly (<10 s) in water, then resuspended in 5 ml of fresh, non-radioactive buffer, which was assayed at intervals for leached (buffer-soluble) [^3^H]Cell_5_-ol. After 6 days, the cellulose was briefly rinsed again in water and assayed for remaining cellulose-bound ^3^H (counting efficiency 7.2%). All data have been corrected for the different counting efficiencies, and show calculated total radioactivity (Bq, from the initial 1100 Bq). The error bars show SD, *n* = 4.

To test the ability of a proposed CCO to hydrogen-bond to almost pure cellulose, we incubated an aqueous solution of [^3^H]Cell_5_-ol in the presence of four types of cellulose. A proportion of the tritium was adsorbed from the solution by binding to the cellulose: rapidly at first and then progressively more slowly. Binding was most rapid, and also most extensive, to filter paper and muslin (Figure 2). In the case of filter paper, ∼61% of the ^3^H had bound within 1 h, and 80% by ∼5 h; little additional binding occurred over the following 2 weeks. Adsorption to Sigmacell and microgranular cellulose was slower and less extensive. Some desorption of ^3^H eventually occurred from muslin and microgranular cellulose; indeed, essentially all the initial binding of [^3^H]Cell_5_-ol to microgranular cellulose had been reversed by ∼2 weeks. In view of the convenience of filter paper and its avid binding of [^3^H]Cell_5_-ol, this form of cellulose was selected for subsequent model experiments.

After 2 weeks’ adsorption, the celluloses were briefly rinsed, then incubated in non-radioactive buffer. Little further desorption occurred over the following 6 days. Finally, the remaining cellulose-bound ^3^H was assayed. The total recovery in the three phases of the experiment was close to 100% with each type of cellulose.

**Figure 3.**
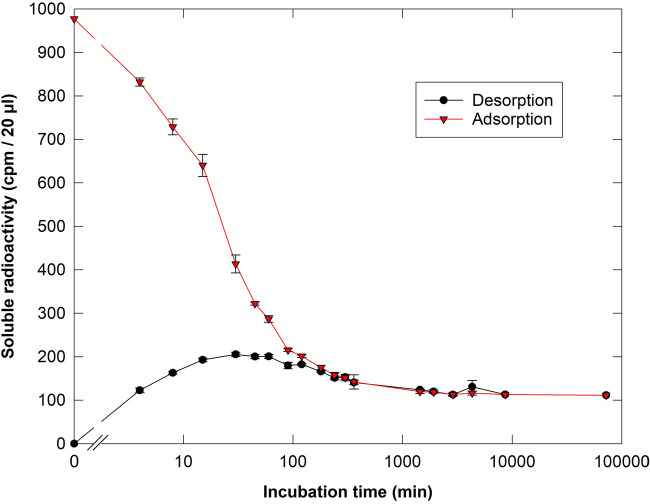
Adsorption/desorption kinetics of [^3^H]Cell_5_-ol to/from cellulosic filter paper. (▾), *Adsorption* of Cell_5_-ol onto filter paper from a 5.0-ml solution in pH 4.75 buffer. (⬤), *Desorption* of Cell_5_-ol (into 5.0 ml of the buffer) off 115 mg filter paper (a 3.5-cm disc of Whatman No. 1) to which it had previously been quickly dried. Note the net desorption from 0 to ∼30 min, followed by partial re-adsorption. For both curves, the beaker containing the 5 ml of buffer was shaken throughout, and the total quantity of ^3^H per beaker was 12.3 kBq (∼244 000 cpm), thus in the adsorption experiment the initial concentration was 61 nM [^3^H]Cell_5_-ol. The error bars show SD, *n* = 3. Results of one of two similar experiments.

In a follow-up experiment on the kinetics of adsorption from aqueous buffer, [^3^H]Cell_5_-ol binding to filter paper approached ∼89% (Figure 3). On the other hand, when a [^3^H]Cell_5_-ol solution was first quickly dried onto filter paper and then incubated in buffer, ∼20% of the ^3^H was gradually *desorbed* (within 30 min) but thereafter about half of that was gradually re-adsorbed. This 20% net desorption shows that some of the oligosaccharide molecules had initially become only weakly attached (during the rapid drying procedure) and were capable of desorbing into solution but then later finding strong binding sites. Thus strong and weak binding sites exist. The final result, however, in both adsorption and desorption experiments, was ∼89% adsorbed.

The above experiments report desorption into a finite volume of water (5 ml). In this situation, desorption could potentially be followed be re-adsorption. To probe *total* desorption, we again dried [^3^H]Cell_5_-ol onto filter-paper (which leads to weak binding; see Figure 3 ‘desorption’ curve) and then washed in an ‘infinite’ volume of water (running tap-water for up to 24 h) such that any transiently desorbed ^3^H would be immediately swept away without the opportunity to re-adsorb. Under these conditions, 70% of the [^3^H]Cell_5_-ol was desorbed from filter-paper (Supplementary Figure S2), which contrasts with the maximum of ∼20% *net* desorption into a finite volume of water where re-adsorption was possible (Figure 3).

### Desorption of a CCO compared with xyloglucan oligosaccharides

In contrast to [^3^H]Cell_5_-ol, ^3^H-labelled oligosaccharides of xyloglucan (which nevertheless possess a cellulose-like backbone) are not expected to be CCOs. They should thus lack to ability to bind cellulose, and, if applied, should rapidly desorb.

To test these predictions, we performed similar experiments with different radiolabelled oligosaccharides and with different washing solutions, and a constant 16-h wash (Table 1). The oligosaccharides had been rapidly dried onto filter-paper and had thus not been given the opportunity to strongly hydrogen-bond. In this experiment, [^3^H]Cell_5_-ol desorption was 53%, 31% and 14% into large volumes of water and 15% and 30% methanol respectively, indicating a kosmotropic (hydrogen-bond-strengthening) effect of methanol. In contrast to [^3^H]Cell_5_-ol, ^3^H-labelled oligosaccharides of xyloglucan showed little tendency to remain cellulose-bound during washing in water. The bigger xyloglucan oligosaccharides (degree of polymerisation ∼14–27) remained partially cellulose-bound in aqueous methanol (kosmotropic effect), but were only negligibly retained in water, even though xyloglucan itself (the polysaccharide) does remain well bound [9,26–28]. This experiment confirms that [^3^H]Cell_5_-ol possesses CCO properties not shared by xyloglucan oligosaccharides.

**Table 1.** Desorption of four oligosaccharides from filter-paper into large volumes of solvents

	^3^H remaining adsorbed on paper (% of unwashed paper)			
	Cell_5_-ol	Glc_12_-based XGO-ols^a^	Glc_8_-based XGO-ols^a^	XXXGol (Glc_4_-based)
Unwashed	100	100	100	100
Water-washed	47.0	3.1	1.6	0.1
15% methanol-washed	68.7	40.3	8.7	0.7
30% methanol-washed	86.2	53.0	12.6	0.8

Each ^3^H-labelled oligosaccharide was dried on to filter paper, which was then washed for 16 h. Water-washing was in flowing tap-water; the methanol washes were 2 × 500 ml. The oligosaccharides were Cell_5_-ol and XGO-ols with Glc_12_-, Glc_8_- or Glc_4_-based backbones (degree of polymerisation ∼21–27, ∼14–18, and 7 respectively).

a XGO-ols, reductively tritiated xyloglucan oligosaccharides.

### Ability of chaotropic agents to desorb firmly bonded CCO from cellulose

To test how firmly [^3^H]Cell_5_-ol adsorbed to cellulose, we allowed this CCO to adsorb to filter paper-discs for 2 weeks as in Figure 2A. The papers were then washed in running tap-water so that only tightly bound oligosaccharide molecules remained on the paper (83% of the supplied [^3^H]Cell_5_-ol). Replicate such papers were then incubated, with gentle shaking, in 5 ml of various potential chaotropic agents. After 24 and 72 h, the release of ^3^H into the solution and (at 72 h) any ^3^H finally remaining on the paper was assayed (Supplementary Figure S3).

As expected, concentrated NaOH very efficiently removed ∼98% of the previously firmly adsorbed CCO from the cellulose. Lower concentrations of alkali were less effective, although even 0.2 M NaOH, a concentration that does not convert cellulose-I to cellulose-II [29–31], solubilised ∼27%. Urea, a widely used chaotropic agent, was moderately effective at 8 M. Cellobiose was particularly effective: even a concentration as low as 0.2 M solubilised >50% of the firmly bonded CCO within 72 h. The structure of cellobiose clearly resembles that of Cell_5_-ol and may thus be expected to act as a competitor of cellulose binding sites.

### Evidence that adsorption of a CCO to cellulose is due to hydrogen-bonding

**Figure 4.**
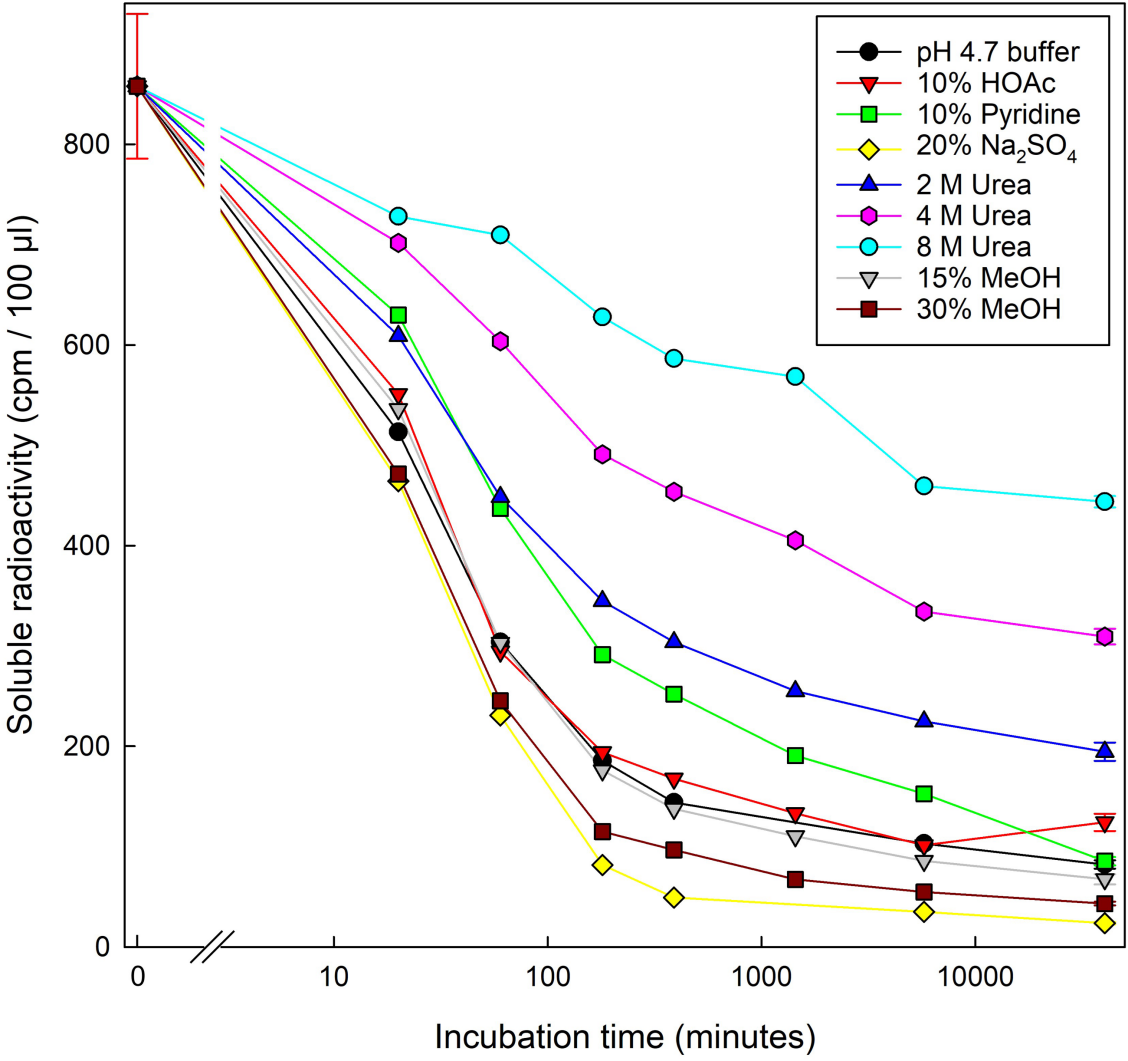
Hydrogen-bonding is responsible for adsorption of [^3^H]Cell_5_-ol onto cellulose. Effect of various solvents (each 5 ml) on the adsorption of 2.27 kBq [^3^H]Cell_5_-ol (initial concentration 11.4 nM) onto 115 mg cellulose (Whatman No. 1 paper). ‘Buffer’ = 100 mM acetate (Na^+^), pH 4.75. All additives except acetic acid (HOAc) and pyridine were dissolved in this buffer. Other details as in Figure 2. Assays for the first (‘0 min’) and final time-point (= 28 days) were replicated, and the error bars show SD, *n* = 6.

To test our assumption that the binding of [^3^H]Cell_5_-ol to filter paper was due to hydrogen bonding, we repeated the adsorption experiment in the presence of various additives (Figure 4). Binding was promoted, both in rate and in extent, by kosmotropes (Na_2_SO_4_ or methanol [32,33]). The most effective example, 1.4 M Na_2_SO_4_, eventually led to 97% adsorption. Conversely, binding was diminished by chaotropes (agents that compete for hydrogen bonding: 2–8 M urea and, weakly, 1.25 M pyridine). Acetic acid (1.75 M) had no effect. These results confirm that the attachment of Cell_5_-ol to cellulose was via hydrogen bonds.

### Cello-oligosaccharides stereospecifically compete with cellopentaitol for adsorption to filter-paper

**Figure 5.**
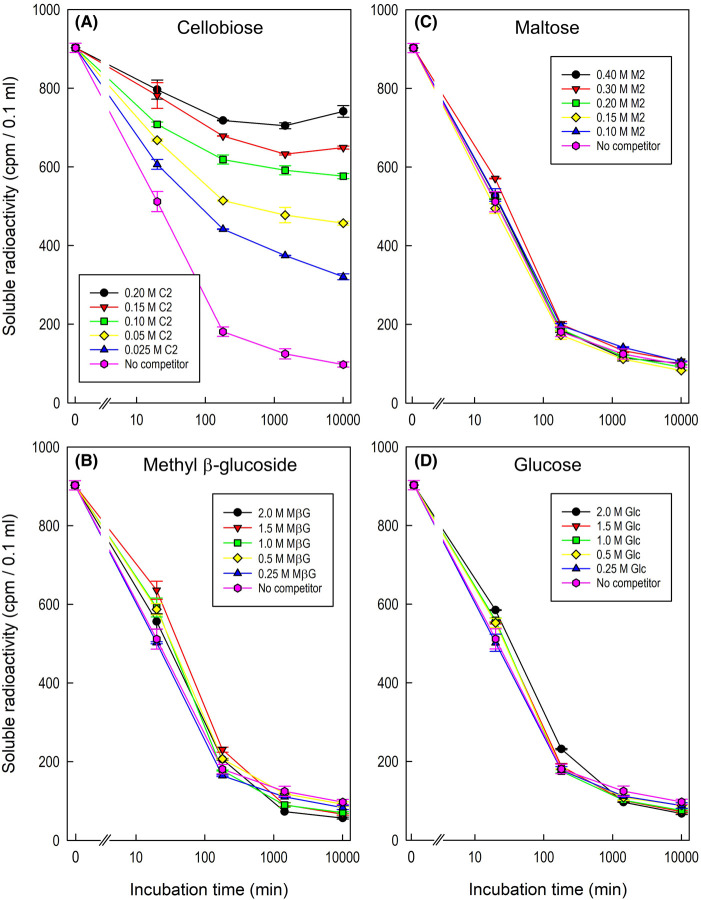
Cellobiose specifically competes with a CCO for binding to cellulose. Adsorption of [^3^H]Cell_5_-ol (2.27 kBq [^3^H]Cell_5_-ol (initial concentration 11.4 nM)) was tested from 5.0 ml of pH 4.75 acetate (Na^+^) buffer onto 115 mg filter-paper in buffer plus various concentrations of four potential competitors: (**A**) C2, cellobiose; (**B**) MβG, methyl β-glucoside; (**C**) M2, maltose; (**D**) Glc, glucose. Error bars represent the range of 2 determinations except in the case of the ‘0 min’ data-point where the error bar represents the mean of 24 independent samples ± SE (*n* = 24). The ‘no competitor’ curve shows the same data on all four graphs. Other details as in Figure 2.

Sugars themselves are not generally regarded as strong chaotropic agents and might therefore be assumed not to mimic urea in inhibiting Cell_5_-ol-cellulose bonding. But interestingly, 200 mM cellobiose [β-Glc-(1→4)-Glc] strongly enhanced desorption (Supplementary Figure S3). In addition, cellobiose competed with the adsorption of Cell_5_-ol to cellulose, half-inhibiting the binding at a concentration of 25–50 mM (Figure 5). Cellobiose's isomer, maltose [α-Glc-(1→4)-Glc], as well as methyl β-d-glucoside and glucose, had no such competing effect, even at much higher concentrations. We conclude that the observed Cell_5_-ol–cellulose interaction occurred via steric complementarity, dependent on the molecular shape of the ligand, and not via generalised hydrogen-bonding. Cellobiose, with a molecular shape comparable to Cell_5_-ol, can thus compete with Cell_5_-ol–cellulose bonding, whereas maltose (identical to cellobiose except for the α-anomerism instead of β-) cannot. Methyl β-glucoside, like Cell_5_-ol, has a β-glucose residue but failed to compete with the Cell_5_-ol–cellulose bonding; thus a β-glucose moiety is necessary but not sufficient for effective competition; at least one neighbouring glucose moiety is also required.

To clarify the roles of *M*_r_ versus α/β-anomerism, we performed similar competition experiments with the two relevant non-radioactive pentasaccharides (cellopentaose and maltopentaose; (1→4)-β- and α-linked Glc_5_ respectively) (Supplementary Figure S4). Adsorption of [^3^H]Cell_5_-ol to filter paper was half-inhibited by ∼25 µM cellopentaose and almost completely inhibited by >100 µM cellopentaose. On the other hand, maltopentaose showed no appreciable competition even at concentrations up to 6500 µM. This confirms the stereospecificity of the competitive effects of non-radioactive oligosaccharides.

**Figure 6.**
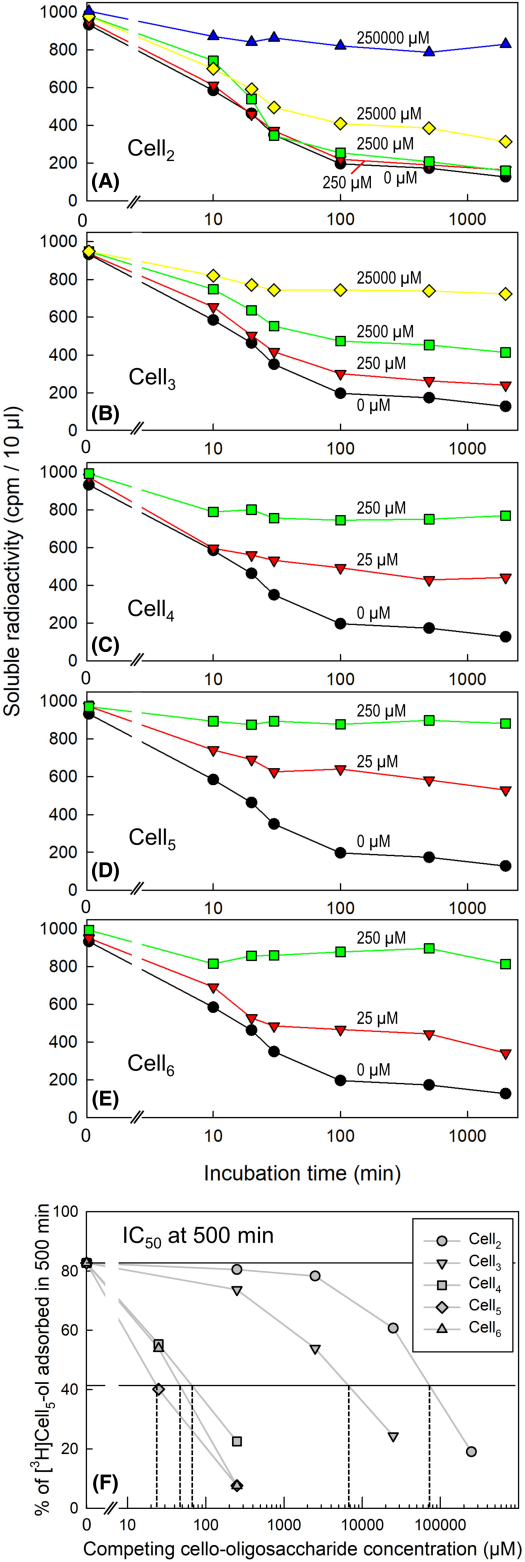
Binding of [^3^H]cellopentaitol to cellulose in the presence of non-radioactive cello-oligosaccharides. Adsorption of [^3^H]Cell_5_-ol (1 kBq, initial concentration 0.63 µM) to 9 mg filter-paper was monitored over time-courses. Oligosaccharides tested as competitors were (**A**) cellobiose, (**B**) cellotriose, (**C**) cellotetraose, (**D**) cellopentaose and (**E**) cellohexaose. Assays in this specific experiment were conducted with *n* = 1, but appropriate concentration ranges had been selected on the basis of preliminary experiments. Graph (**F**) shows the approximate IC_50_ values (concentrations that half-inhibited the adsorption of [^3^H]Cell_5_-ol) at the 500-min time points in graphs (**A**) to (**E**). The two horizontal lines indicate the % binding at 500 min in the absence of competitors and half that % binding. The dashed vertical lines indicate the approximate IC_50_ values.

Likewise, to clarify the importance specifically of *M*_r_ we made a comparison of all the water-soluble cello-oligosaccharides (non-radioactive Cell_2_ to Cell_6_) and their concentrations causing 50% inhibition of [^3^H]Cell_5_-ol binding (IC_50_). There was a large difference between Cell_2_ (IC_50_ ≈ 25–50 mM) and Cell_5_ (IC_50_ ≈ 25 µM) (Figure 6). Thus competition was strongly dependent on the chain-length of the cello-oligosaccharide competitor, with Cell_5_ being 1000–2000× more effective than Cell_2_. The approximate IC_50_ values were: Cell_2_, 70 mM; Cell_3_, 7 mM; Cell_4_, 70 µM; Cell_5_, 25 µM; Cell_6_, 50 µM (Figure 6F). Thus, Cell_3_ was ∼10× more effective than Cell_2_. Between Cell_3_ and Cell_4_ there was a dramatic increase in effectiveness, with Cell_4_ being ∼100× more effective than Cell_3_. There was little difference between Cell_4_, Cell_5_ and Cell_6_.

### Effect of pH on adsorption of CCO to cellulose

**Figure 7.**
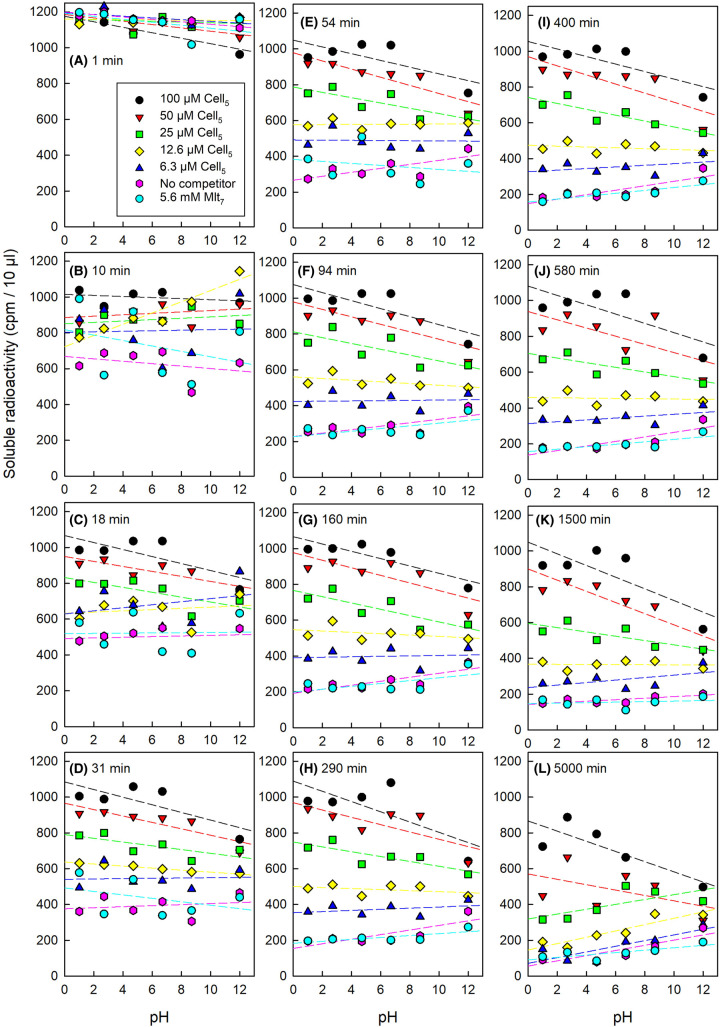
Effect of pH on the binding of [^3^H]Cell_5_-ol to cellulose with and without non-radioactive cellopentaose. The graphs show the ability of [^3^H]Cell_5_-ol (1 kBq, initial concentration 0.63 µM) to bind to filter paper in buffers at pH 1–12 in the presence of 0–100 µM non-radioactive cellopentaose (Cell_5_), and, for comparison, 5.6 mM maltoheptaose (Mlt_7_). Samples were tested for remaining non-adsorbed ^3^H after 0–5000 min (graphs **A–L**, respectively). The key in graph (**A**) also applies to all the other graphs. Linear regressions (SigmaPlot) were fitted to each dataset (dashed lines). The statistical significance of the apparent gradients is described in Supplementary Table S1.

Figure 4 indicates that a high pH (10% pyridine; pH 9.7) somewhat diminishes the cellulose adsorption of [^3^H]Cell_5_-ol compared with pH 4.75 (buffer) and 2.2 (10% acetic acid). That experiment was conducted in the absence of non-radioactive competing oligosaccharides. We next defined the effects of a broader range of pH values on [^3^H]Cell_5_-ol adsorption in the presence and absence of Cell_5_ (0–100 µM) and, as a control, maltoheptaose (5600 µM) (Figure 7).

As in previous experiments, high Cell_5_ concentrations strongly competed with the adsorption of tracer concentrations of [^3^H]Cell_5_-ol, regardless of pH and incubation time (Figure 7). Intriguingly, however, a high pH *promoted* the extent of adsorption achieved at high cello-oligosaccharide concentrations (25–100 µM Cell_5_). On the other hand, a high pH *diminished* relative adsorption at low cello-oligosaccharide concentrations (6.3 and 0 µM Cell_5_, including 0 µM Cell_5_ plus 5600 µM maltoheptaose), agreeing with the effect of pyridine (pH 9.7) noted in Figure 4. At the intermediate concentration of 12.5 µM Cell_5_, pH had little consistent effect. The above trends (especially for the low and zero Cell_5_ concentrations) became more pronounced at longer incubation times. The trends are summarised by colour-coding in Supplementary Table S1.

Taking into account the total quantity of cello-oligosaccharide adsorbing (µmol Cell_5_-ol + Cell_5_, assuming that these two pentasaccharides behave identically), we made some unexpected observations. A high pH ultimately allowed the adsorption of a greater *quantity* of cello-oligosaccharides even though it inhibited the adsorption of trace quantities of [^3^H]Cell_5_-ol at low µM concentrations. In Supplementary Figure S5, the data are re-plotted in terms of nmol of total pentasaccharide bound per mg cellulose. This shows an enhancement by high pH of binding capacity. The maximum binding capacity observed (∼1 nmol/mg) corresponds to ∼0.8 µg pentasaccharide per mg cellulose.

### Binding of CCO to cellulose is related to the amount of paper present

This experiment explored the relationship between CCO–cellulose binding and the weight of cellulose supplied (or the volume of buffer used). At 0 µM Cell_5_, halving the weight of cellulose had little effect, supporting the idea that the cellulose was then far from saturated by pentasaccharide (Supplementary Figure S6). However, at 25 and 250 µM cellopentaose, halving the weight of cellulose substantially diminished CCO binding. For example, at 25 µM Cell_5_, ∼43% of the ^3^H bound to 9 mg paper and ∼22% bound to 4.5 mg paper (Supplementary Figure S6a,b), indicating that the cellulose was approaching saturation with pentasaccharide.

A similar conclusion was reached when the volume of pentasaccharide solution was reduced (at constant cellulose weight). For example, at 25 µM pentasaccharide, ∼43%, 19% and 9% was bound when 200, 400 and 800 µl of solution, respectively, was supplied to 9 mg of cellulose (Supplementary Figure S6b–d). The experimental system developed thus enables a ‘titration’ of the amount of exposed cellulose.

### Applying [^3^H]Cell_5_-ol to explore the exposure of microfibrils in wild-type and transgenic cotton

Note: Since our cotton fibres contained measurable non-cellulosic polymers in addition to cellulose, we refer to them as ‘cellulose-rich material’. However, when discussing the adsorption of ^3^H-labelled CCOs to cotton fibres we refer to ‘cellulose binding’ since the non-cellulosic components are thought not to contribute to CCO binding.

**Figure 8.**
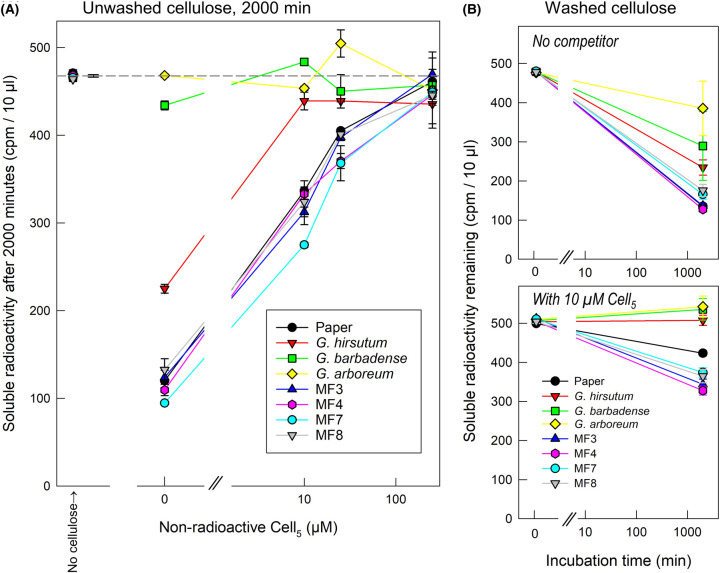
Adsorption of a CCO to cotton celluloses. Cotton cellulose (10 mg) was incubated in 200 µl buffer containing 1 kBq [^3^H]Cell_5_-ol (1 kBq, initial concentration 0.63 µM) plus 0–250 µM non-radioactive Cell_5_. (**A**) Unwashed cellulose with various concentrations of competing non-radioactive Cell_5_; constant incubation time 2000 min. The left-hand symbols show measurements with no added cellulose, thus effectively time-zero controls; the dashed grey line shows these controls’ mean value ± SD (*n* = 3). (**B**) Washed cellulose with 0 or 10 µM non-radioactive Cell_5_ and 0 or 2000 min incubation times. Washing (when applied) was performed sequentially in 90% dimethylsulphoxide, 75% ethanol, 1% aqueous Triton X-100 and 100% acetone, followed by re-drying. The cellulose samples used were Whatman No. 1 filter-paper and cotton ‘fibres’ from wild-type *G. hirsutum*, *G. arboreum* and *G. barbadense* plus four transgenic lines (MF2, MF4, MF7 and MF8; all *G. hirsutum* plants transformed with *WRI1* from *G. barbadense*). The data are from one of two similar (but not identical) experiments, which led to the same conclusions.

Using the techniques developed above, we explored the surface accessibility of cellulose within cotton ‘fibres’ from wild-type *G. arboreum*, *G. hirsutum* and *G. barbadense* and four lines of *G. hirsutum* transformed with *WRI1* of *G. barbadense*. In one experiment, the incubation time was kept constant at 2000 min, and [^3^H]Cell_5_-ol binding was measured in the presence of 0, 10, 25 and 250 µM competing non-radioactive Cell_5_ (Figure 8A). Almost no ^3^H was bound to *G. barbadense* and *G. arboreum* even in the absence of Cell_5_ competitor. In contrast, *G. hirsutum* bound ∼50% of the ^3^H at 0 µM cellopentaose; adsorption was completely eliminated by the presence of ≥10 µM Cell_5_. In all the transgenic *G. hirsutum* specimens (and in filter-paper), 70–80% of the [^3^H]Cell_5_-ol was adsorbed in the absence of competitor, decreasing to 30–40% at 10 µM Cell_5_ and to 10–20% at 25 µM. Changes in cellulose behaviour may have been caused by altered carbon flow away from fatty-acid metabolism towards sugar biosynthesis.

A possible explanation for the lack of ^3^H binding to some wild-type cotton fibres could have been the presence of waxes, pectins or other substances ensheathing the cellulose of the fibres. We therefore re-tested cotton fibres after washing in a series of solvents and a detergent (Figure 8B). Washing did permit subsequent measurable adsorption by all cotton samples, though washed *G. arboreum* cellulose-rich material remained only weakly able to bind [^3^H]Cell_5_-ol, indicating that its cellulose was least accessible to the surrounding solution. The cellulose-rich material of the wild-types was very susceptible to competition by 10 µM non-radioactive Cell_5_, indicating a limited capacity for pentasaccharide binding (in µmol/mg cellulose-rich material) even after removal of waxes etc.

### Characterising surface accessibility of different celluloses by cellulase digestion

To further validate the CCO binding method for probing cellulose-rich material surface accessibility, we tested an alternative strategy: measuring the susceptibility of cellulose samples to digestion by endo-1,4-β-d-glucanase (cellulase).

Filter-paper was most susceptible to enzymic hydrolysis (Supplementary Figure S7), agreeing with its high accessibility to [^3^H]Cell_5_-ol. Similarly, buffer-washed *G. arboreum* cellulose-rich material was least susceptible to enzymic hydrolysis (Supplementary Figure S7), corroborating the [^3^H]Cell_5_-ol adsorption data which had indicated that its cellulose-rich material was least accessible to the surrounding solution. Intermediate results were obtained with wild-type *G. barbadense* and *G. hirsutum*, and also with the four transgenic lines. The agreement between the results of the two unrelated methods — [^3^H]Cell_5_-ol binding and glucanase digestion — supports their validity.

### Non-cellulosic polysaccharides present in cotton fibres

**Figure 9.**
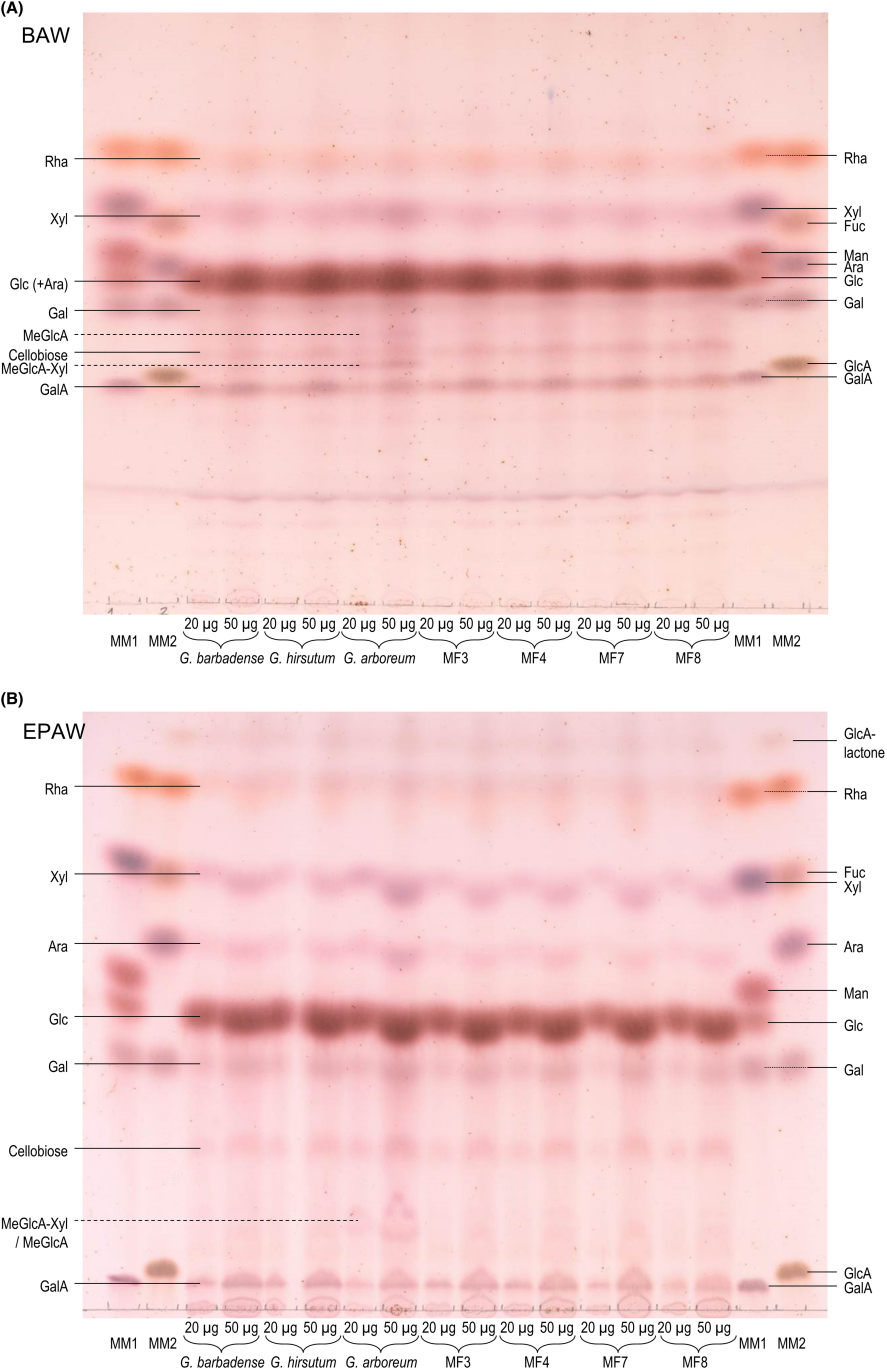
Cell-wall matrix composition of cotton fibre samples. Seven types of cotton fibre were washed sequentially in 90% dimethylsulphoxide, 75% ethanol, 1% Triton X-100 and pure acetone, then dried, and portions (each 10 mg) heated in 2 M TFA. The products obtained from 20 and 50 µg of dried fibres were analysed by TLC on silica-gel in (**A**) butan-1-ol/acetic acid/water (BAW, 4:1:1 by volume, with two ascents) or (**B**) ethyl acetate/pyridine/acetic acid/water (EPAW, 6:3:1:1 by volume, with two ascents) and stained with thymol. Each image shows one typical example out of three replicate chromatograms. MM1 and MM2 = marker mixtures (each sugar 1 µg except GlcA lactone, which was a contaminant in the GlcA). Right-hand labels refer to marker sugars, left-hand ones to sugars found in the cotton hydrolysates. Dashed lines refer to sugars not definitively identified: MeGlcA, 4-*O*-methylglucuronic acid; MeGlcA-Xyl, 4-*O*-methyl-α-d-glucuronosyl-(1→2)-xylose.

The cotton samples studied in the present work were cotton fibres, which, although very rich in cellulose, are expected also to contain small amounts of non-cellulosic cell-wall-matrix polysaccharides [34]. We investigated possible compositional differences between the seven cotton fibre specimens by hydrolysis in trifluoroacetic acid (TFA) followed by thin-layer chromatography (TLC). TFA efficiently hydrolyses matrix polysaccharides but not cellulose. Two TLC systems were used, differing in their ability to cause uronic acids to migrate (Figure 9).

All cotton fibre samples produced glucose as the major monosaccharide plus smaller amounts of cellobiose, probably mainly because of the ability of TFA to gradually hydrolyse a small proportion of the cellulose. In addition, however, they also produced various non-cellulosic sugars: of these, xylose (a hemicellulose component) predominated and was more abundant in *G. arboreum* than in the other six samples. We also detected four monosaccharides typical of pectin: galacturonic acid, rhamnose, galactose and arabinose (arabinose is also present in some heteroxylan hemicelluloses). Most of these pectic monosaccharides were found in similar quantities in all seven fibre types (but slightly more arabinose in *G. arboreum*). Interestingly, *G. arboreum* yielded two unique sugars that were essentially undetectable in all the other specimens, putatively 4-*O*-methyl-d-glucuronic acid and the relatively acid-resistant dimer 4-*O*-methyl-α-d-glucuronosyl-(1→2)-xylose, suggesting a relatively high content of glucurono(arabino)xylan [35]. This high glucurono(arabino)xylan content correlates with, and may explain, the low cellulose accessibility noted in *G. arboreum* fibres in the earlier experiments.

## Discussion

### Mechanism of cello-oligosaccharide–cellulose bonding

The aim of this work was to characterise a radiolabelled CCO and its ability to adsorb to cellulose, thus potentially giving a simple, sensitive and quantitative way of titrating ‘naked’ cellulose surfaces which are of interest in plant cell-wall physiology and commercial cellulose preparations such as cotton fibres. We confirmed that [^3^H]Cell_5_-ol quickly and avidly adsorbs to pure cellulose. For example, filter-paper adsorbed 40% and >80% of aqueous 5 nM [^3^H]Cell_5_-ol within ∼1 and ∼20 h respectively. The cellulose-rich material of muslin (cheesecloth) adsorbed slightly less total [^3^H]Cell_5_-ol, slightly more slowly. Two other commercial celluloses (microgranular and ‘Sigmacell’) adsorbed far less. The filter-paper and muslin released relatively little of the initially bonded [^3^H]Cell_5_-ol upon washing in water, indicating avid bonding. Sigmacell in contrast quickly released a high proportion of any initially bonded [^3^H]Cell_5_-ol. All these samples are almost pure cellulose; thus the differences between them in CCO adsorption and desorption were dependent on the biophysical nature of the microfibrils.

We showed that within a single cellulose type (filter-paper) there are at least two types of [^3^H]cellopentaitol bonding: weak bonds formed rapidly but were reversible upon washing in water, and strong bonds formed slowly but were not reversible in water. Almost all (∼98%) of the ‘strong’ bonds could, however, be broken in concentrated alkali, which is expected to confer negative charges on ‘neutral’ carbohydrates such as cellulose and cello-oligosaccharides such that they electrostatically repel one another. Urea (8 M), a well-known chaotropic agent, also released some (∼30%) of the strongly adsorbed [^3^H]Cell_5_-ol within 1 day, indicating that the attachment of [^3^H]Cell_5_-ol to cellulose was via hydrogen-bonds. Particularly effective was cellobiose, which cleaved about half the ‘strong’ bonds within 1–3 days. Cellobiose is not generally regarded as an effective chaotropic agent, and thus apparently worked by virtue of its ability to mimic the specific molecular shape of [^3^H]Cell_5_-ol. Thus hydrogen bonds that are dependent on the specific structural complementarity between [^3^H]Cell_5_-ol and cellulose are concluded to be responsible for the observed adsorption.

We compared the above effects of additives on [^3^H]Cell_5_-ol *desorption* with tests of their effects on [^3^H]Cell_5_-ol *adsorption* to cellulose. In agreement with the above, 8 M urea delayed the progress and lessened the final adsorption of [^3^H]Cell_5_-ol to filter-paper. Predictably, kosmotropes (hydrogen-bond-strengthening agents: 1.4 M Na_2_SO_4_ or 30% methanol), enhanced adsorption. Also in agreement with the desorption experiments, non-radioactive cello-oligosaccharides competitively inhibited the adsorption of [^3^H]Cell_5_-ol to filter paper, whereas malto-oligosaccharides did not (even at much higher concentrations). This confirms the key role of structural complementarity between cellulose and Cell_5_-ol. We tested all existing water-soluble cello-oligosaccharides (Cell_2–6_), and found the effectiveness of differently sized competitors to be Cell_6_ ≈ Cell_5_ ≈ Cell_4_ >> Cell_3_ > Cell_2_. Thus, cello-oligosaccharides larger than the trisaccharide were extremely efficient at blocking [^3^H]Cell_5_-ol–cellulose bonding.

By assuming that cellulose-binding by [^3^H]Cell_5_-ol (the reduced pentasaccharide) resembles that of Cell_5_ (the non-radioactive, reducing pentasaccharide), we gained insight into the quantity of total cello-pentasaccharide that can bind to cellulose. The maximum adsorption, i.e. under conditions where the cellulosic surfaces were saturated, was ∼1 nmol of total pentasaccharide per mg cellulose (≈0.8 µg/mg) (Supplementary Figure S5). This indicates that only in the order of 0.1% of any given segment of cellulose chain was capable of bonding the cello-pentasaccharides. Some of the remaining cellulose segments would have been sequestered within the structure of a microfibril, but a majority must have been incapable of binding the pentasaccharides because of structural features such as crystallinity. Other factors remaining constant, the quantity adsorbed was proportional to the mass of cellulose supplied, so the methodology is capable of ‘titrating’ exposed binding sites within cellulose.

The data also revealed that mild alkali-pretreatment (pH 8.4–12) of cellulose increased its capacity for pentasaccharide binding, presumably by exposing otherwise sequestered segments of cellulose. We suggest that at a low cello-pentasaccharide concentration, at which almost 100% of the supplied oligosaccharide can readily bind to cellulose, the predominant effect of high pH is to act as a mild chaotropic agent, interfering in hydrogen-bonding. However, at high concentrations, where 100% binding can never be approached, the total binding achieved is enhanced by the ability of high pH to open up the architecture of the microfibrils, thus exposing some cellulose chains that would otherwise have been hidden from soluble oligosaccharides present in the surrounding solution. This conclusion underlines the value of CCO adsorption as a strategy to define the accessibility of cellulosic surfaces to the surrounding solution.

### Cotton fibres differ in cellulose accessibility

Of seven types of cotton ovular trichomes (‘fibres’ of commerce) tested, wild-type *G. arboreum* fibres were least capable of adsorbing [^3^H]cellopentaitol. This result shows that different cotton fibres differ in the surface accessibility of their cellulose and suggests ensheathment of a proportion of their microfibrillar surfaces. The unique properties of *G. arboreum* fibres were supported by their high resistance to digestion by endo-1,4-β-d-glucanase (cellulase). It is known that highly crystalline (hence relatively inaccessible) cellulose is most resistant to cellulase digestion [36]. Another possible agent tending to exclude enzymes in some cellulose samples is lignin, which is indeed reported to retard digestion by cellulase [36,37]. The agreement between the results of the two unrelated methods ([^3^H]Cell_5_-ol binding and cellulase digestion) supports their validity. However, we prefer the radiolabelled CCO binding method as it is quicker and simpler to quantify than the cellulase method.

The above unique features of *G. arboreum* fibres were correlated with the presence in that cotton species of high levels of glucuronoarabinoxylan, a hemicellulose (non-cellulosic polysaccharide) of the cell wall, which was potentially responsible for the ensheathing of cellulose.

### Potential future applications of the CCO adsorption methodology

The biological interest of this work focuses on the fact that cellulosic microfibrils in plant cell walls are largely ensheathed, and probably tethered, by hydrogen-bonded hemicelluloses. Ensheathing may vary developmentally as hemicelluloses are peeled to enable cell expansion [11]. We characterised a simple method to quantify ensheathed *versus* naked cellulosic surfaces based on the ability to adsorb a radiolabelled ‘cellulose-complementary oligosaccharide’ (CCO), [^3^H]cellopentaitol. Mapping and quantifying naked cellulose surfaces is of interest in two situations:

(a) monitoring areas of naked cellulose that are believed to occur in the cell walls of living plant cells and to increase in amount when the cell grows. Fast- and slow-growing plant cells (e.g. in dark- and light-grown seedlings respectively) are predicted to differ in exposed cellulosic surface areas as hemicelluloses are stripped off, ‘Velcro-like’ [11], potentially favoured by expansin proteins [12], during cell expansion. The CCO method could be applied in such systems with the aim of giving the first clear experimental evidence for expansin action in the walls of fast-growing cells.
(b) describing and explaining the properties of commercially important cellulose samples such as pulp, textiles (e.g. cotton and linen), medical swabs and cellulose-based thickeners (e.g. Curran® [12]). Measurements of accessible cellulose area may correlate with these products’ functional quality [38]. Cellulose has a vast array of commercial applications, and the accessible (‘naked’) surface area of the cellulose fibres is often crucial in determining their desirable properties, including their ability to interact with neighbouring molecules ranging from water [39] to fish oil [40], paper [41], viscosity enhancers [42], foodstuffs [43,44], plastics and enzymes [36,37]. The ability to probe cellulose for ‘naked’ surfaces by use of CCOs is a beneficial addition to the available analytical methodology.

The basis of the methodology characterised here is the ability of CCOs to hydrogen-bond firmly to cellulose surfaces. CCOs bind in a complementary manner, comparable to the well-documented hydrogen-bonding between the complementary chains of a DNA double helix. The CCO–cellulose bonding can be sensitively and quantitatively monitored if the CCOs have been pre-labelled with a suitable tag. The methodology can in principle be used to characterise this bonding (e.g. its specificity and permanence), and to explore the surface accessibility of cellulosic structures in biologically and biotechnologically important situations.

The specific CCO explored here was a radioactively labelled oligosaccharide. Alternative labelling methods could include sulphorhodamine labelling, with localisation achieved by fluorescence microscopy [45] or high-throughput dot-blot screening [46]. Further variations on the methodology could potentially include other tagged CCOs, such Cell_6_-ol (cellohexaose is the largest fragment of cellulose that is appreciably water-soluble at neutral pH), and oligosaccharides of β-1,4-mannan and β-1,4-xylan, which possess a similar 3-dimensional structure to cellulose. Of these, at least [^3^H]Cell_6_-ol and [^3^H]Xyl_6_-ol spontaneously hydrogen-bond to cellulose from aqueous solution and remain partially bound when washed in water [16]; thus they are CCOs and can potentially be used as probes for ‘naked’ cellulosic surfaces as in Figure 1.

## Conclusions

In conclusion, the adsorption to cellulose of [^3^H]cellopentaitol, and potentially other CCOs, is a simple, sensitive and quantitative way of titrating ‘naked’ cellulose surfaces in diverse botanical and industrial samples. The adsorption occurs by hydrogen-bonding and requires a strict complementarity of structure between the CCO and cellulose. For example, cello-oligosaccharides serve as CCOs, whereas isomeric malto-oligosaccharides do not. A minimum of four glucose residues is necessary for maximal hydrogen bonding of cello-oligosaccharides to cellulose. The quantity of cello-pentasaccharide adsorbed is proportional to the weight of cellulose tested, thus the method enables a titration of binding sites. The enzymic digestibility of various cellulose-rich samples and their accessibility to [^3^H]cellopentaitol adsorption led to similar conclusions concerning the surface exposure of the microfibrils, supporting the validity of these two unrelated methods; however, we prefer the CCO binding method as it is quicker and simpler to quantify. CCO binding was used to monitor the accessibility of several cotton (*Gossypium*) genotypes. *G. arboreum* cellulose was least accessible to the surrounding solution, correlating with (and possibly due to) its relatively high content of ensheathing glucurono(arabino)xylan. Future applications of the CCO adsorption methodology include exploring the properties of diverse cellulose-rich samples, both in biotechnologically important cellulose-rich preparations (such as cotton fibres, medical swabs and cellulose-based thickeners) and in the plant cell wall (either *in vivo* during cell expansion or post-harvest).

## Materials and methods

### Materials

The celluloses tested in model experiments were Whatman No. 1 filter-paper, microgranular cellulose (Sigma C-6413), Sigmacell Type 100 (sold for cellulose column chromatography; Sigma S-3755), and muslin (cheesecloth). Although not 100% cellulose, we refer to these (very) cellulose-rich materials as ‘cellulose’

Whatman No. 1 paper is essentially pure cellulose (>98%; manufacturer's information) produced from cotton linters and largely freed of cutin and hemicelluloses. It has a relatively high Segal CI (obtained by X-ray diffraction), variously reported as 83.5% [47], 74% [48] and 86% [49]. Similarly, Thygesen et al. [50] found CI ≈ 83% for another type of filter paper (Frisenette No. 165). In contrast, Kaschuk and Frollini [51] reported ‘filter paper’ (manufacturer not specified) to have CI ≈ 63%, although this filter paper contained 19.7% hemicelluloses (much higher than in Whatman No. 1) and its CI abruptly rose to a stable 79% after only 4 minutes' treatment with a mixture of ‘cellulases’ (containing ‘hemicellulases’). It seems likely that CI ≈ 80% is a realistic estimate for filter papers in general and the Whatman No. 1 used here in particular.

Sigma C-6413 microgranular cellulose (synonyms: cellulose powder, cotton linters) is reported to have CI ≈ 85% [52]. Sigmacell 100 is reported to have a relatively low CI of 57.4% (from X-ray diffraction patterns collected in transmittance mode) or 70.6% (in reflectance mode) [53]. Muslin is a loosely woven fabric of cotton (see next paragraph).

Later experiments employed cellulose-rich cotton fibres. Raw cotton fibres are very rich in cellulose. Reported CI values for raw cotton linter are 87% [54], 91% [55] and 64% [56]. Cotton linter pulp had CI ≈ 70%, this value being unaffected by ‘cooking’ treatments in alkaline H_2_O_2_ which nevertheless strongly diminished the degree of polymerisation of the cellulose [57]. The cotton fibres used in the present paper were from wild-type *G. hirsutum* (cultivar FH 490; long staple), *G. arboreum* (FDH 786; short staple) and *G. barbadense* (Bar 14/5; extra long staple) and from four transgenic lines of *G. hirsutum* (MF2, MF4, MF7 and MF8) transformed with *WRI1* of *G. barbadense*. Cotton was grown outdoors near Lahore, Pakistan, during the monsoon months of May to August, known as the *kharif* period. Seeds were sown in the field following the standard protocols, giving a continuous supply of fibres for the experiments. All fibre samples were taken when fully mature (∼50 days post-anthesis).

Cell_3_, Cell_4_, Cell_5_ and Cell_6_ (cellotriose, cellotetraose, cellopentaose and cellohexaose) were from Megazyme (Bray, Ireland). Other non-radioactive reagents were from Sigma–Aldrich (Poole, Dorset, U.K.). [^3^H]Cellopentaitol (Cell_5_-ol; specific activity ∼40 or ∼8 MBq/µmol) was from EDIPOS (https://fry.bio.ed.ac.uk/edipos.html). Reductively tritiated xyloglucan oligosaccharides were also from EDIPOS: the Glc_12_- and Glc_8_-based oligosaccharides were ∼30 MBq/µmol, and the Glc_4_-based heptasaccharide (XXXGol) was ∼900 MBq/µmol. Cellulase [(endo β-1-4-glucanase, incapable of digesting xyloglucan] from *Aspergillus niger* was from Megazyme.

### CCO adsorption studies

An aliquot (1.0 or 1.1 kBq unless otherwise indicated) of [^3^H]Cell_5_-ol was dried *in vacuo* and then redissolved 0.2 or 5.0 ml of buffer [routinely 83 or 100 mM acetate (counterion Na^+^ or pyridinium^+^), pH 4.75, containing 0.5% chlorobutanol (volatile antimicrobial agent)] and containing other components as specified in Figure legends. Dry cellulose-rich material (routinely 90 or 115 mg for 5-ml solutions or 9 mg for 0.2-ml) was then added to the solution and incubated with gentle agitation at 20°C. Samples of the free solution were taken at timed intervals, and assayed for soluble radioactivity.

In the experiment where pH 1–12 was tested, the buffers were: 120 mM HCl (pH ∼1), 150 mM citrate (Na^+^, pH 2.7 and 6.7), 160 mM acetate (Na^+^, pH 4.7), 150 mM TAPS (Na^+^, pH 8.7), and 150 mM phosphate (Na^+^, pH 12.0).

### Assay of radioactivity

Radiolabelled solutions were mixed with 10 volumes of OptiPhase HiSafe 3 scintillation fluid (Perkin Elmer, Waltham, U.S.A.) and assayed for ^3^H in a Beckman LS5000 scintillation-counter. Solid samples (e.g. pieces of filter-paper or chromatography paper) were dried, placed in 2 ml of water-immiscible scintillant (Gold Star; Meridian Biotechnologies Ltd) and assayed in the same counter.

### Cellulase digestion

Dry cotton fibres (10 mg) were suspended in 1.0 ml 166 mM acetate (pyridinium^+^) buffer, pH 4.75, containing 0.5% chlorobutanol and incubated at 20°C with gentle agitation. After 1 h, the suspension was bench-centrifuged and 50 µl of clear supernatant was taken as an enzyme-free control. The cellulose was resuspended in the remaining 950 µl of buffer, 1 U of cellulase in 10 µl of the same buffer was added, and incubation was continued. After 7 and 14 days, a 50-µl sample of supernatant taken as before. Portions (5 µl) of each supernatant sample were dried onto a piece of silica-gel TLC plate, alongside a dilution series of glucose, and the spots were thymol-stained (dipped in 0.5% thymol and 5% H_2_SO_4_ in ethanol [58], dried for ∼15 min, then heated in an oven at 105°C for 15 min) and quantified from scans.

### Analysis of TFA-hydrolysed non-cellulosic cotton-fibre polysaccharides

Triplicate 10-mg samples of cotton fibre were washed sequentially with 90% dimethylsulphoxide (DMSO), 75% ethanol, 1% Triton X-100 and pure acetone, then re-dried. DMSO is an aprotic, amphipathic solvent expected to solubilise most low-*M*_r_ phytochemicals, including inorganics. Ethanol (75%) is another highly effective solvent for low-*M*_r_ phytochemicals, its use being recognised in the standard practice of preparing ‘ethanol-insoluble residues’ during cell-wall isolation. Triton X-100 is a neutral detergent, helping to solubilise oils. And acetone, besides removing other remaining lipids and relatively hydrophobic phytochemicals, expedites drying of the cellulose-rich samples. Hydrolysis was conducted in 1 ml 2 M TFA at 120°C for 1 h. After cooling and centrifugation, the supernatant was freed of TFA by drying *in vacuo*. The residue was redissolved in the original volume of 0.5% aqueous chlorobutanol, and 2 and 5 µl was analysed by TLC (i.e. the hydrolysis products from 20 and 50 µg fibres). TLC was on 20 × 20-cm silica-gel plates in ethyl acetate/pyridine/acetic acid/water (EPAW, 6:3:1:1 by volume) and (butan-1-ol/acetic acid/water (BAW, 4:1:1 by volume). The TLC plates were stained with thymol as above.

### Paper chromatography of oligosaccharides

Non-radioactive oligosaccharides (each 50 µg) or a trace (2.35 kBq) of [^3^H]Cell_5_-ol were applied to Whatman No. 1 chromatography paper and run in the four solvents indicated in Supplementary Figure S1. Non-radioactive sugars were stained with AgNO_3_ [59] and [^3^H]Cell_5_-ol was quantified by scintillation-counting of 1-cm strips.

## Data Availability

All data relevant to this manuscript are provided within the paper as tables, Figures and supplementary file. We have not included any structural/crystallographic data (for either macromolecular structures or small molecules), protein and nucleic acid sequence data other than what are already published on universally accessible websites, functional genomics and molecular interactions/proteomics/metabolomics data, computational models, or genetics data (genetic polymorphisms; genotype data).

## Open Access

Open access for this article was enabled by the participation of The University of Edinburgh in an all-inclusive *Read & Publish* agreement with Portland Press and the Biochemical Society under a transformative agreement with JISC.

## Abbreviations

CCO: cellulose-complementary oligosaccharide
DMSO: dimethylsulphoxide
Cell_2–6_: cellobiose to cellohexaose
Cell_5_-ol: borohydride-reduced cellopentaose
CI: crystallinity index
cpm: counts per minute
IC_50_: concentration causing 50% inhibition of [^3^H]Cell_5_-ol binding
TFA: trifluoroacetic acid
TLC: thin-layer chromatography
XXXG: heptasaccharide of xyloglucan (Xyl_3_·Glc_4_)
