# Supplementary material for: Using a cellulose-complementary oligosaccharide as a tool to probe exposed cellulosic surfaces in cotton fibres and growing plant cell walls
Source: Biochem J. 2024 Sep 13;481(18):1221–40. doi: 10.1042/BCJ20240296 (PMC11555694; doi:10.1042/BCJ20240296)
Supplement: Supplementary Material [file BCJ-481-1221-s1.pdf]

Using a cellulose-complementary oligosaccharide as a tool to probe exposed cellulosic surfaces in cotton fibres and growing plant cell walls

Mahnoor Imran[1,*] Lenka Franková[1], Uzma Qaisar[2] and Stephen C. Fry[1]

[1]The Edinburgh Cell Wall Group, Institute of Molecular Plant Sciences, The University of Edinburgh, Edinburgh EH9 3BF, UK

[2]School of Biological Sciences, University of the Punjab, Lahore, Pakistan.

*Present address: School of Biological Sciences, University of the Punjab, Lahore, Pakistan

Supplementary file

CONTENTS

Figure S1: Mobility of reducing gluco-oligosaccharides and [^{3}H]Cell$_5$-ol on paper chromatography

Figure S2. Desorption of a CCO from filter-paper into an 'infinite' volume of water

Figure S3 Resistance of firmly hydrogen-bonded CCO to elution by various chaotropic agents.

Figure S4. Cellopentaose, but not maltopentaose, competes with [^{3}H]Cell$_5$-ol for binding to cellulose.

Figure S5. Effect of pH and time on absolute quantity of pentasaccharide bound to cellulose.

Figure S6. Effect of cellulose weight, buffer volume and total CCO concentration on adsorption of pentasaccharide to cellulose.

Figure S7. Susceptibility of various celluloses to enzymic hydrolysis.

Table S1. Correlations between pH and remaining unbound radioactivity.

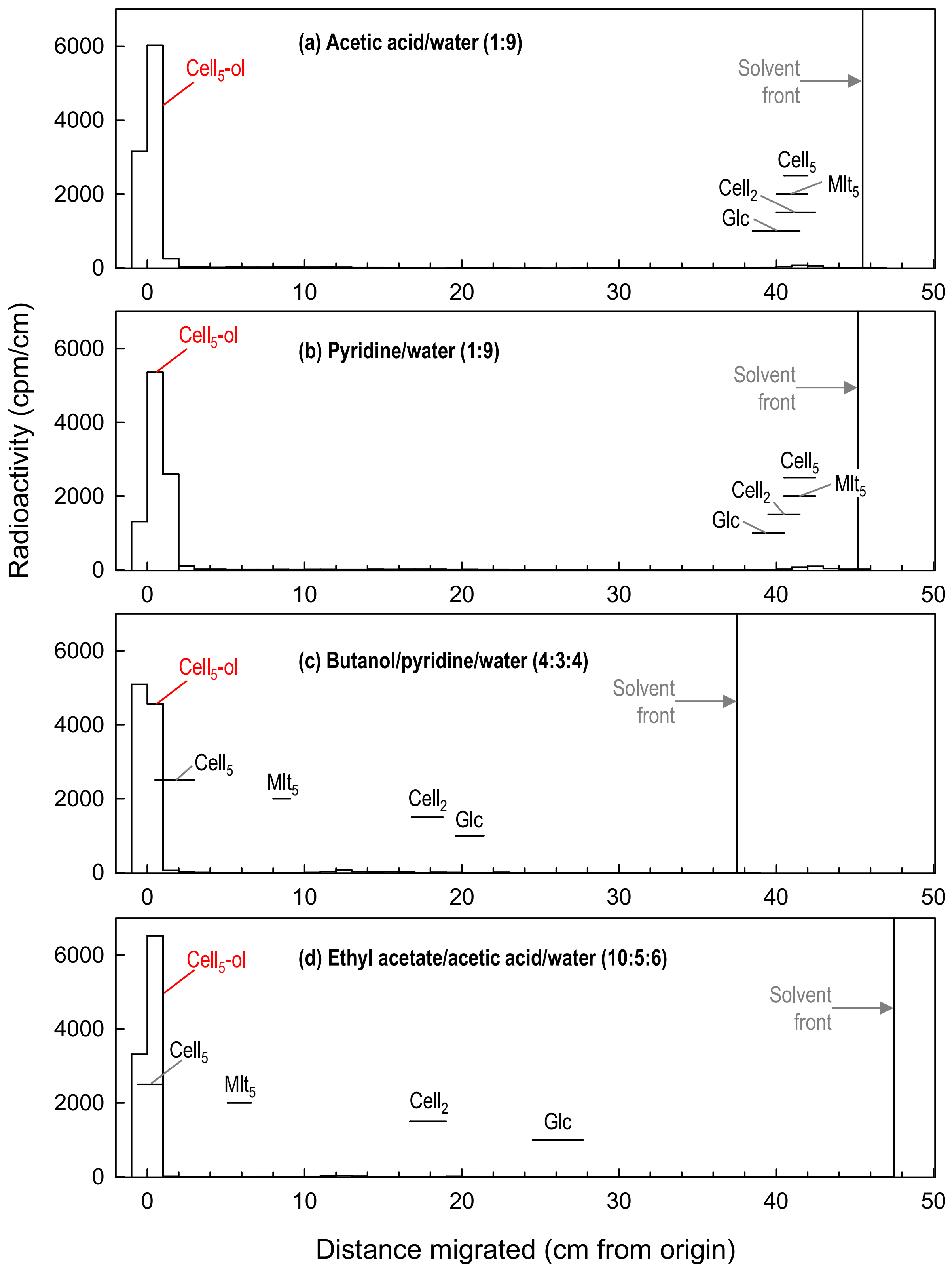

Figure S1: Mobility of reducing gluco-oligosaccharides and [^{3}H]Cell$_5$-ol on paper chromatography

Samples were applied to Whatman No. 1 chromatography paper. Solvents (a) and (b) were run for 8 h; (c) and (d) for 16 h. The positions of non-radioactive external marker sugars (glucose, cellobiose, cellopentaose, maltoheptaose), stained with $AgNO_3$, are indicated by horizontal lines. The [^{3}H]Cell$_5$-ol was quantified by scintillation-counting as shown by the histograms (counts per minute per strip). Solvent compositions are given by volume.

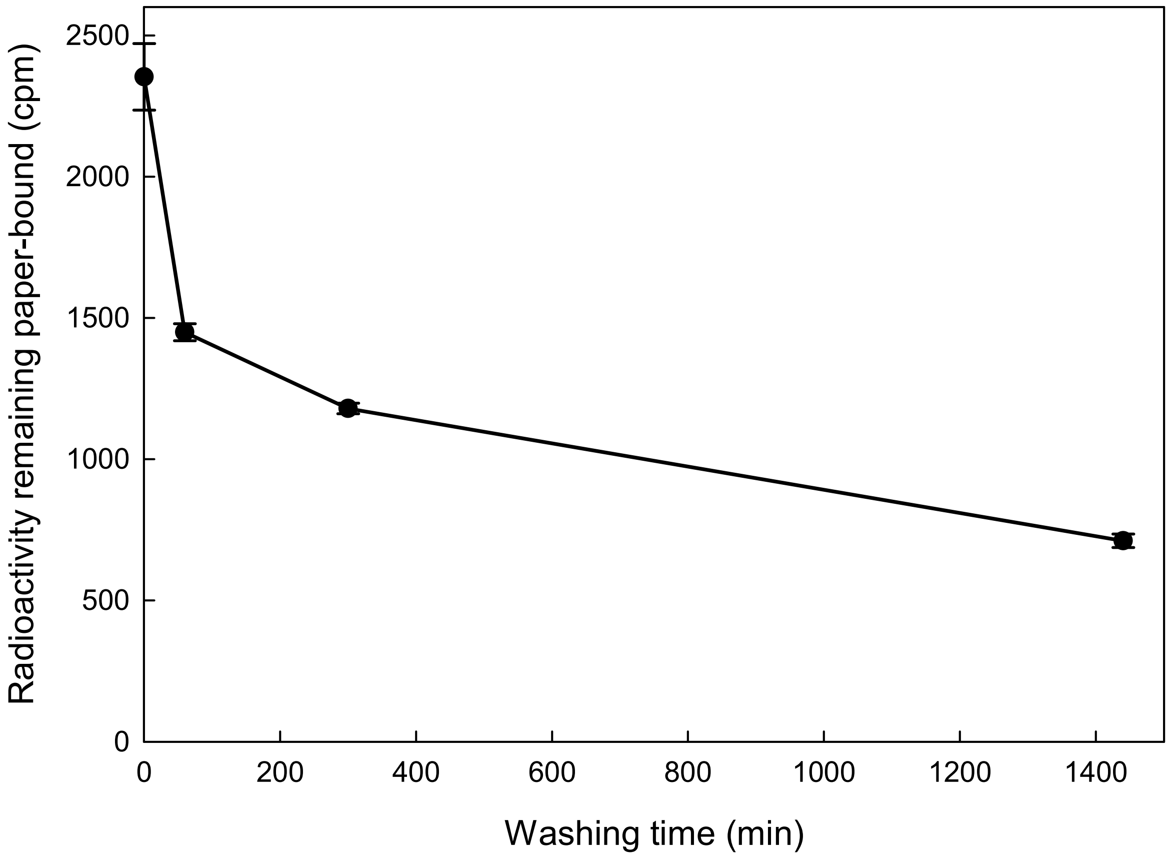

Figure S2. Desorption of a CCO from filter-paper into an 'infinite' volume of water

[^{3}H]Cell$_5$-ol (454 Bq; 11.4 pmol) was quickly dried onto 115 mg filter paper, which was then washed in running tap-water for up to 24 h. Radioactivity remaining on replicate papers, removed at intervals, was assayed. Error bars show SE; n = 5 papers.

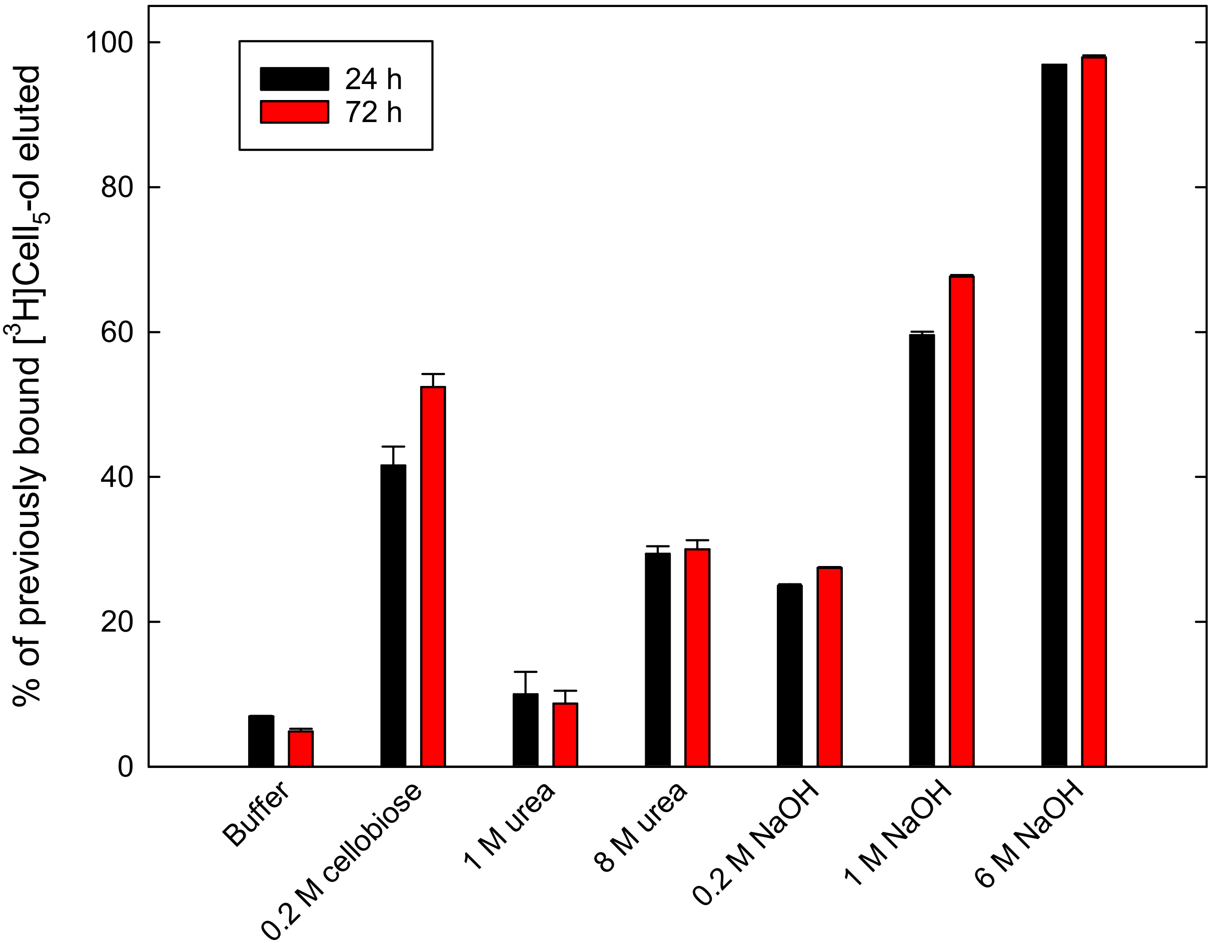

Figure S3 Resistance of firmly hydrogen-bonded CCO to elution by various chaotropic agents.

[^{3}H]Cell$_5$-ol (initial concentration 5 nM) was allowed to adsorb to fourteen 90-mg discs of filter paper in 5 ml buffer for 14 d, then (without a drying step) the paper was washed in running tap-water for a further 2 d, removing weakly-bonded molecules of the CCO. Replicate papers were then incubated with gentle shaking in 5 ml of the seven solutions listed on the *x*-axis. Samples (0.5 ml) of the solutions were taken at 24 and 72 h, neutralised if necessary, diluted to 2.0 ml with water, and assayed for released ^{3}H. Finally, the papers were washed for 16 h in running tap-water, and assayed for remaining bound ^{3}H. From the data, the % elution of [^{3}H]Cell$_5$-ol by the potential chaotropes was determined. Error bars show the range for two identical papers.

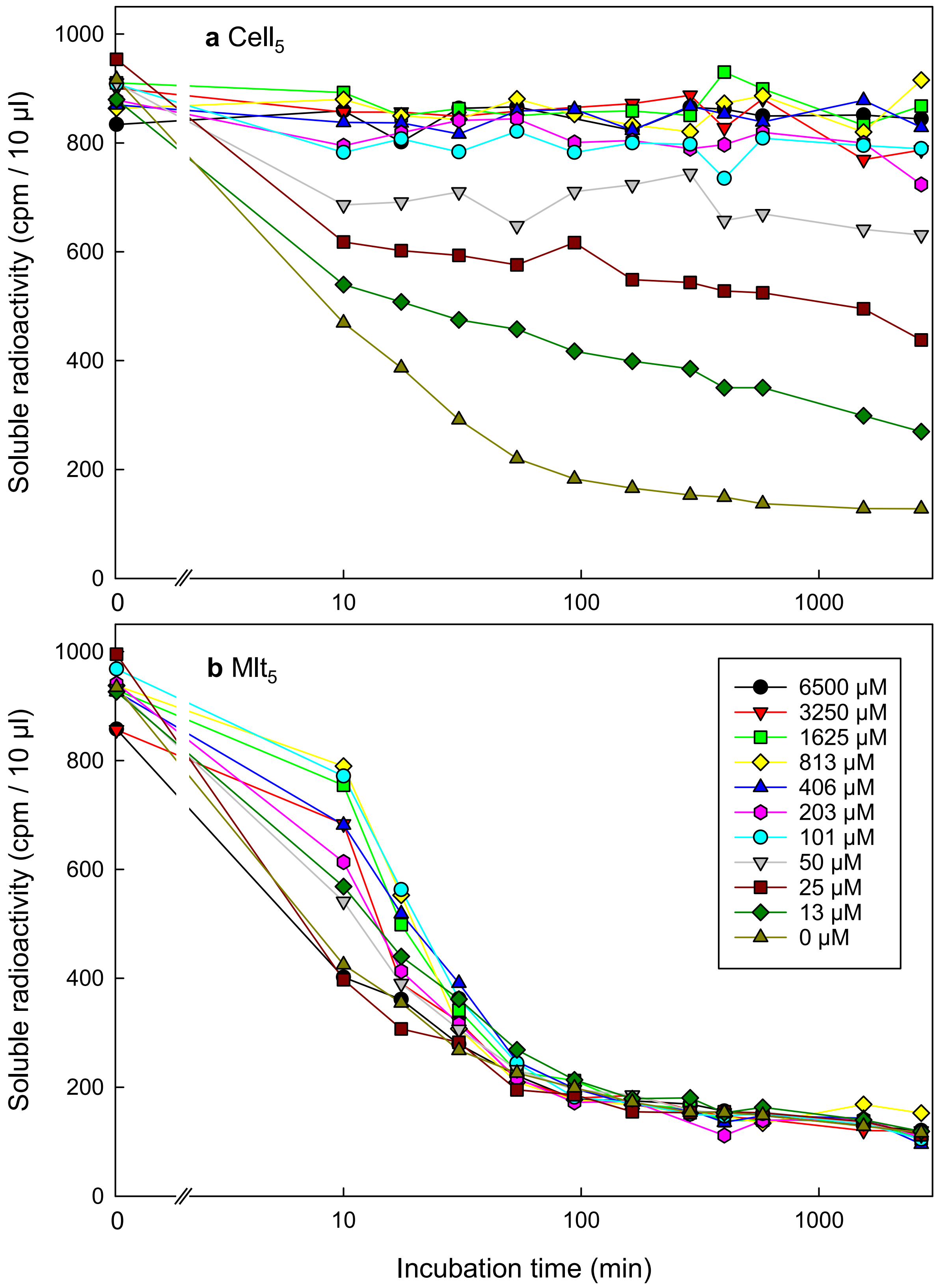

Figure S4. Cellopentaose, but not maltopentaose, competes with [^{3}H]Cell$_5$-ol for binding to cellulose.

Adsorption of [^{3}H]Cell$_5$-ol [1 kBq, initial concentration 0.63 µM] was tested from 200 µl of pH 4.75 buffer onto 9 mg filter-paper in the presence of various concentrations of (a) cellopentaose or (b) maltopentaose. Soluble [^{3}H]Cell$_5$-ol remaining in the solution was assayed after various incubation times. Other details as in Figure 2. The pentasaccharide concentrations shown in graph (b) also apply to (a). Data are from one of two similar experiments.

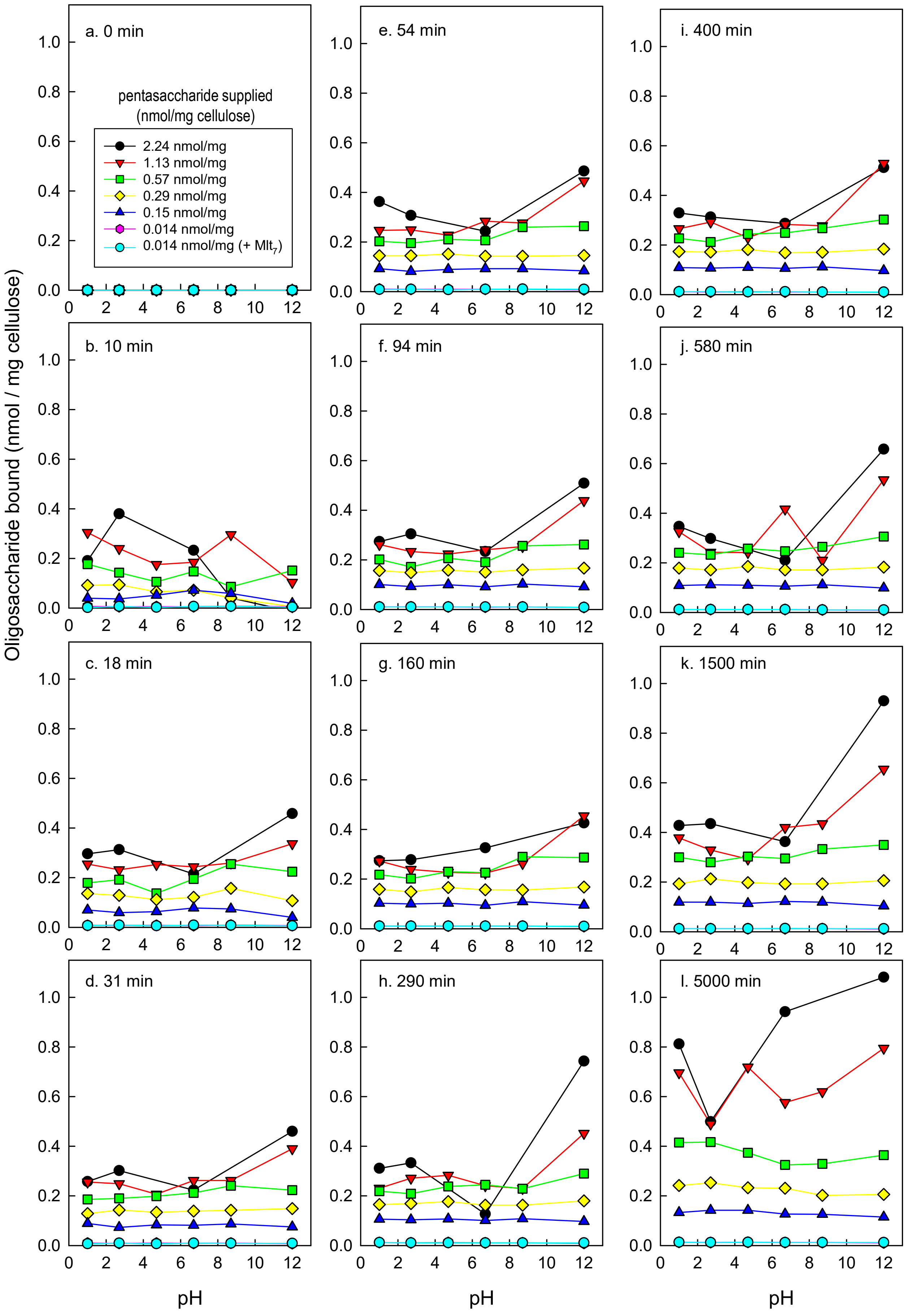

Figure S5. Effect of pH and time on absolute quantity of pentasaccharide bound to cellulose.

The graphs show the ability of total cello-pentasaccharide ([^{3}H]Cell$_5$-ol + Cell$_5$, assumed to behave identically) to bind to filter paper, buffered at pH 1–12. For comparison, a trace of [^{3}H]Cell$_5$-ol in the presence of 5.6 mM maltoheptaose was tested (cyan symbols) and the data are indistinguishable from a trace of [^{3}H]Cell$_5$-ol alone (magenta symbols, eclipsed by the cyan). Binding was estimated by disappearance of soluble ^{3}H after various incubation times, 0–5000 min (graphs a–l, respectively), when pentasaccharide was supplied in the quantities indicated by the different symbols (see key in Figure a). Data are re-plotted from Figure 7.

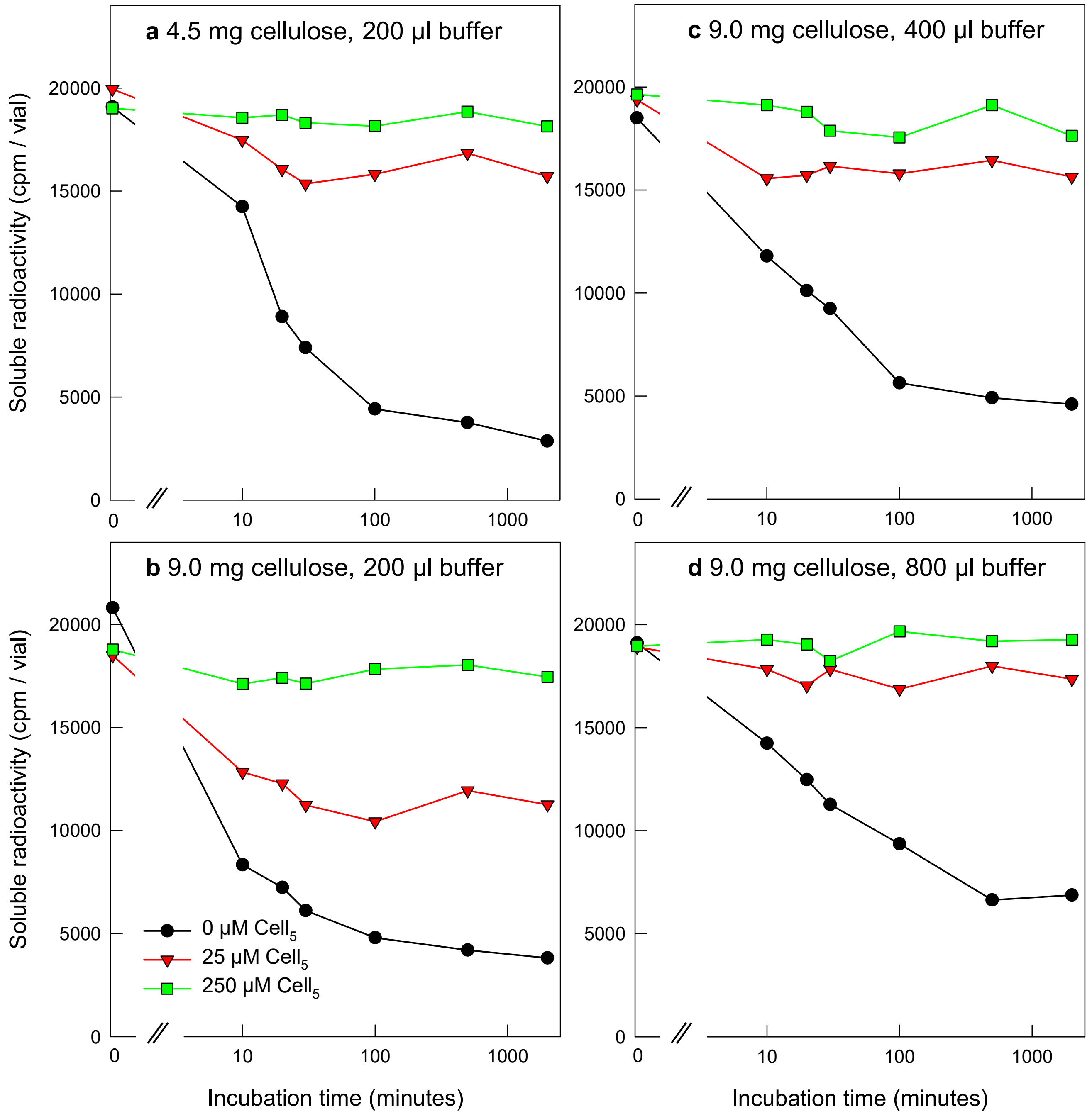

Figure S6. Effect of cellulose weight, buffer volume and total CCO concentration on adsorption of pentasaccharide to cellulose.

Suspensions contained 1 kBq [^{3}H]Cell$_5$-ol (1 kBq,) in 200–800 µl of buffer (thus initial Cell$_5$-ol concentration 0.63, 0.32 or 0.16 µM) supplemented with 0, 25 or 250 µM non-radioactive Cell$_5$. At time 0, filter-paper (cellulose) was added and the remaining free soluble ^{3}H was assayed at intervals.

(a) 4.5 mg cellulose, 200 µl buffer;

(b) 9.0 mg cellulose, 200 µl buffer (as in previous experiments);

(c) 9.0 mg cellulose, 400 µl buffer (i.e., ratio as in (a));

(d) 9.0 mg cellulose, 800 µl buffer.

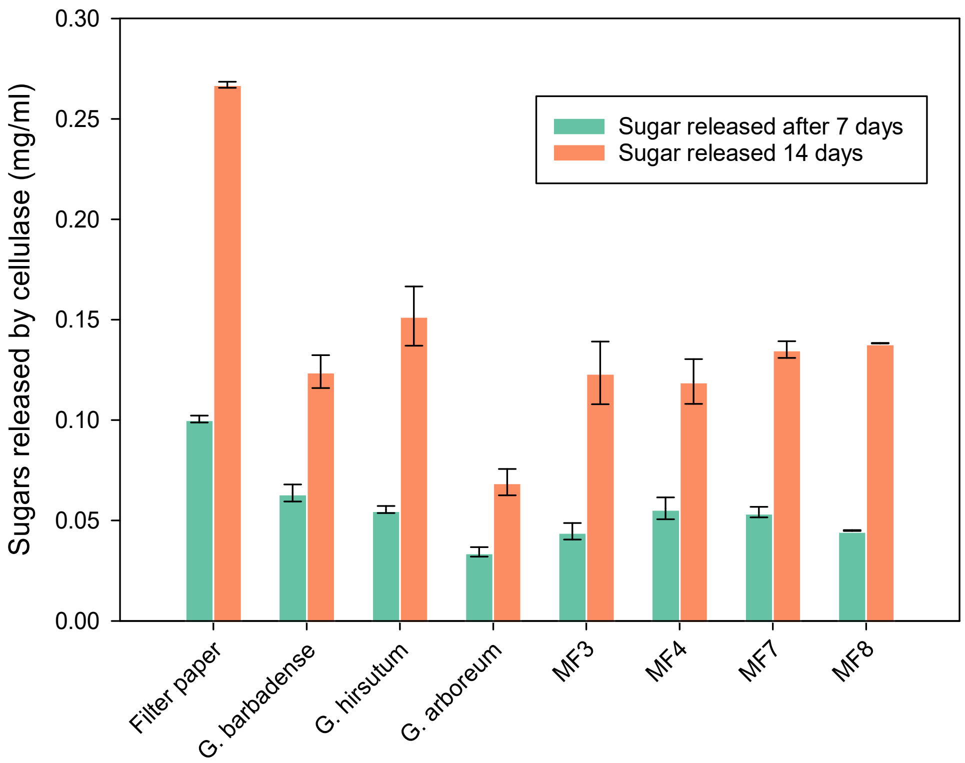

Figure S7. Susceptibility of various celluloses to enzymic hydrolysis.

Each cellulose sample (9 mg) was incubated with 1 U of endo-1,4-β-D-glucanase (cellulase) in 1.0 ml of 83 mM acetate (pyridinium⁺) buffer, pH 4.75, at 20°C on a mixing wheel for 7–14 days. Solubilised carbohydrate was then assayed by a thymol staining method, with glucose as the standard. Controls with enzyme but no cellulose, or with cellulose but no enzyme, were run alongside; no sugars released in controls. Prior to this experiment, each 9-mg cotton sample was washed with, sequentially, 90% DMSO, 75% ethanol, 1% Triton X-100 and pure acetone (14 ml each for 2–16 h) followed by drying. Bars indicate SD (n = 3). The data are from one of two similar experiments, which led to comparable conclusions.

Table S1. Correlations between pH and remaining unbound radioactivity.

DF = 5

Deep blue: negative correlation with p<0.02
Blue: negative correlation with p<0.05
Pale blue: trending negative correlation with p<0.10
Unshaded: no significant correlation
Pale yellow: trending positive correlation with p<0.10
Yellow: positive correlation with p<0.05
Orange: positive correlation with p<0.02

Figures in bold: correlation coefficient (r)
Other figures: gradient of line of best fit, ± SE

Incubation time →	10 min	18 min	31 min	54 min	94 min	160 min	290 min	400 min	580 min	1500 min	5000 min
↓ Competing oligosaccharide											
**100 µM Cell5**	**0.32**<br>-3.0 ± 5.0	**0.74**<br>-19.5 ± 10.3	**0.77**<br>-21.3 ± 10.2	**0.71**<br>-18.8 ± 10.7	**0.80**<br>-22.4 ± 9.9	**0.87**<br>-20.5 ± 6.7	**0.72**<br>-28.7 ± 15.9	**0.80**<br>-21.0 ± 9.2	**0.74**<br>-26.0 ± 13.7	**0.78**<br>-32.5 ± 14.9	**0.83**<br>-28.5 ± 11.2
**50 µM Cell5**	**0.29**<br>3.9 ± 6.4	**0.82**<br>-13.9 ± 4.9	**0.85**<br>-17.9 ± 5.4	**0.88**<br>-22.7 ± 6.0	**0.80**<br>-20.8 ± 7.9	**0.77**<br>-21.1 ± 8.8	**0.74**<br>-20.4 ± 9.3	**0.81**<br>-25.4 ± 9.3	**0.66**<br>-22.9 ± 13.2	**0.88**<br>-31.2 ± 8.4	**0.48**<br>-15.0 ± 13.6
**25 µM Cell5**	**0.33**<br>3.87 ± 5.6	**0.72**<br>-13.6 ± 6.6	**0.72**<br>-10.5 ± 5.1	**0.80**<br>-14.8 ± 5.6	**0.73**<br>-16.2 ± 7.6	**0.80**<br>-17.6 ± 6.6	**0.81**<br>-13.5 ± 4.9	**0.87**<br>-16.5 ± 4.8	**0.80**<br>-13.0 ± 4.8	**0.77**<br>-12.0 ± 4.9	**0.70**<br>13.5 ± 6.9
**12.5 µM Cell5**	**0.95**<br>31.3 ± 5.0	**0.18**<br>3.5 ± 9.3	**0.98**<br>-5.7 ± 0.5	**0.05**<br>0.3 ± 2.7	**0.54**<br>-4.6 ± 3.6	**0.40**<br>-3.8 ± 4.3	**0.40**<br>-2.9 ± 3.3	**0.34**<br>-2.3 ± 3.2	**0.11**<br>-0.8 ± 3.7	**0.05**<br>-0.3 ± 3.0	**0.91**<br>17.7 ± 3.9
**6.3 µM Cell5**	**0.04**<br>1.5 ± 19.4	**0.30**<br>8.5 ± 13.7	**0.06**<br>0.9 ± 7.6	**0.04**<br>-0.5 ± 6.3	**0.07**<br>0.8 ± 5.5	**0.10**<br>1.3 ± 6.0	**0.36**<br>3.1 ± 4.1	**0.41**<br>4.5 ± 5.0	**0.56**<br>5.2 ± 3.9	**0.54**<br>6.9 ± 5.4	**0.82**<br>15.8 ± 5.6
**None**	**0.32**<br>-6.8 ± 9.9	**0.14**<br>1.8 ± 6.5	**0.20**<br>2.9 ± 7.7	**0.72**<br>11.1 ± 5.4	**0.69**<br>9.7 ± 5.1	**0.82**<br>11.1 ± 3.9	**0.79**<br>12.8 ± 4.9	**0.81**<br>12.4 ± 4.5	**0.83**<br>12.7 ± 4.2	**0.79**<br>4.3 ± 1.7	**0.84**<br>14.4 ± 4.7
**5600 µM Mlt7**	**0.30**<br>-15.1 ± 24.1	**0.02**<br>0.6 ± 13.2	**0.38**<br>-9.6 ± 11.8	**0.24**<br>-5.6 ± 11.1	**0.60**<br>7.6 ± 5.1	**0.59**<br>7.9 ± 5.4	**0.75**<br>5.4 ± 2.4	**0.84**<br>8.1 ± 2.6	**0.79**<br>6.9 ± 2.6	**0.22**<br>1.4 ± 3.1	**0.77**<br>6.8 ± 2.8

The data are calculated from Fig. 7, which shows the effect of pH on the binding of $[^3H]Cell_5$-ol to cellulose in the presence and absence of potentially competing oligosaccharides. The Table reports the estimated gradients (±SE) of the lines of best fit. The correlation coefficient (*r*) indicates the statistical significance of the apparent gradient. Cells in the Table are shaded to draw attention to negative gradients (shades of blue) and positive gradients (shades of yellow). A negative gradient indicates that a high pH promotes binding; a positive gradient indicates that a low pH promotes binding. The intensity of shading indicates the statistical significance of the apparent gradient.

Each scatter plot in Fig. 7 has six datapoints and thus 5 degrees of freedom, and the probability (*p*) that the apparent gradient is due to fluke is deduced from *r*. The strong correlation in the 10-min sample with 12.5 µM $Cell_5$ is unexplained. Otherwise, there is a clear tendency for positive gradients at low or zero $Cell_5$ concentrations (especially at later time-points) and for negative gradients at high $Cell_5$ concentrations. Note that the trends for 5600 µM maltoheptaose (not a cellulose-binding oligosaccharide) resemble those obtained in the absence of any oligosaccharide.
